# Human Milk Micronutrients and Child Growth and Body Composition in the First 2 years: A Systematic Review

**DOI:** 10.1016/j.advnut.2023.06.005

**Published:** 2023-06-13

**Authors:** Sarah M. Reyes, Meredith (Merilee) Brockway, Joann M. McDermid, Deborah Chan, Matthew Granger, Rebecca Refvik, Karanbir K. Sidhu, Suad Musse, Caroline Monnin, Larisa Lotoski, Donna T. Geddes, Fyezah Jehan, Patrick Kolsteren, Lindsay H. Allen, Daniela Hampel, Kamilla G. Eriksen, Natalie Rodriguez, Meghan B. Azad

**Affiliations:** 1 Manitoba Interdisciplinary Lactation Centre, Children’s Hospital Research Institute of Manitoba, University of Manitoba, Winnipeg, Manitoba, Canada; 2 Department of Pediatrics and Child Health, University of Manitoba, Winnipeg, Manitoba, Canada; 3 Consultant, Charlottesville, VA, USA; 4 Department of Epidemiology, Biostatistics, and Occupational Health, McGill University, Montréal, Québec, Canada; 5 Department of Food and Human Nutritional Sciences, University of Manitoba, Winnipeg, Manitoba, Canada; 6 Neil John Maclean Health Sciences Library, University of Manitoba, Winnipeg, Manitoba, Canada; 7 School of Molecular Sciences, the University of Western Australia, Perth, Western Australia, Australia; 8 Department of Pediatrics & Child Health, Aga Khan University, Karachi, Pakistan; 9 Department of Food Safety and Food Quality, Ghent University, Ghent, Belgium; 10 Department of Nutrition, University of California, Davis, Davis, CA, USA; 11 United States Department of Agriculture, Western Human Nutrition Research Center, Agriculture Research Service, Davis, CA, USA; 12 Department of Nutrition, Exercise and Sports, Faculty of Science, University of Copenhagen, Copenhagen, Denmark

**Keywords:** human milk, infant, anthropometry, micronutrients, calcium, zinc, body composition, growth, stunting, lactation

## Abstract

Human milk (HM) provides a plethora of nutritional and non-nutritional compounds that support infant development. For many compounds, concentrations vary substantially among mothers and across lactation, and their impact on infant growth is poorly understood. We systematically searched MEDLINE, Embase, the Cochrane Library, Scopus, and Web of Science to synthesize evidence published between 1980 and 2022 on HM components and anthropometry through 2 y of age among term-born infants. Outcomes included weight-for-length, length-for-age, weight-for-age, body mass index (in kg/m^2^)–for–age, and growth velocity. From 9992 abstracts screened, 144 articles were included and categorized based on their reporting of HM micronutrients, macronutrients, or bioactive components. Micronutrients (vitamins and minerals) are reported here, based on 28 articles involving 2526 mother-infant dyads. Studies varied markedly in their designs, sampling times, geographic and socioeconomic settings, reporting practices, and the HM analytes and infant anthropometrics measured. Meta-analysis was not possible because data were sparse for most micronutrients. The most-studied minerals were zinc (15 articles, 1423 dyads) and calcium (7 articles, 714 dyads). HM iodine, manganese, calcium, and zinc concentrations were positively associated with several outcomes (each in ≥2 studies), whereas magnesium (in a single study) was negatively associated with linear growth during early lactation. However, few studies measured HM intake, adjusted for confounders, provided adequate information about complementary and formula feeding, or adequately described HM collection protocols. Only 4 studies (17%) had high overall quality scores. The biological functions of individual HM micronutrients are likely influenced by other HM components; yet, only 1 study analyzed data from multiple micronutrients simultaneously, and few addressed other HM components. Thus, available evidence on this topic is largely inconclusive and fails to address the complex composition of HM. High-quality research employing chronobiology and systems biology approaches is required to understand how HM components work independently and together to influence infant growth and to identify new avenues for future maternal, newborn, or infant nutritional interventions.

**Statements of significance**

Our work comprehensively synthesizes evidence regarding associations between individual HM micronutrients and child anthropometrics among healthy, term-born infants. This manuscript is part of a larger systematic review that also investigated HM macronutrients and bioactive components, reported in separate manuscripts (PROSPERO: CRD42020187350).

## Introduction

The health consequences of suboptimal breastfeeding, especially in the first 6 mo of life, may result in as many as 1.4 million child deaths annually and account for an estimated 10% of the total burden of diseases in childhood globally [1]. More favorable outcomes observed in breastfed infants are attributed, in part, to the plethora of nutritional and non-nutritional components of human milk (HM) [[2], [3], [4], [5]]—many of which vary substantially in concentration diurnally, over the course of lactation and among women [2,6]. Despite a marked increase in HM research in recent decades, major gaps remain in our understanding of how HM components influence infant growth.

Infant and young child growth outcomes are general health indicators that predict social development, educational attainment, and later health [1,7,8]. Proper nutrition is essential for achieving healthy growth, especially during periods of rapid growth such as infancy. A hallmark of inadequate nutrition is stunting, which affects nearly a third of children and is a major cause of morbidity and mortality globally [1]. In a recent pooled analysis of 31 longitudinal cohorts comprising 62,993 children in low-resource settings, the highest incidence of stunting onset occurred between birth and 3 mo, when HM is recommended as the sole source of nutrition [9]. This suggests that HM components critically influence growth in early infancy–although it remains important to understand the continued role of HM after complementary foods are introduced since breastfeeding is recommended through 2 y or longer [10]. Accordingly, our objective was to synthesize evidence on the associations between HM components and child anthropometry in the first 2 y. Results were organized into the following categories: micronutrients (vitamins and minerals), macronutrients (lipids, proteins, and digestible carbohydrates), and bioactive components (e.g., cytokines, hormones, and nondigestible carbohydrates). Here we report the evidence for micronutrients.

Micronutrients play a critical role in the growth and development of children [11,12]. Although it is well documented that deficiency in some micronutrients, such as zinc (Zn), iron (Fe), and vitamin A, may result in poor childhood growth [1], knowledge about the specific relationships between HM micronutrients and infant and young child growth is incomplete [13]. These relationships are difficult to discern, in part, because of the multiple factors that influence infant nutritional status. For example, infants whose mothers are nutritionally replete are born with reserves of some micronutrients (e.g., Zn, Fe, and vitamin B12) [4], which may prevent infant deficiency even if HM concentrations are low. Nevertheless, even in well-nourished mothers, genetics, diet, health status, and other environmental factors can influence the concentration and bioactivity of micronutrients in their milk and, consequently, infant status [4]. Recent reviews have summarized the state of the evidence on HM micronutrients, reporting on how their concentrations change throughout lactation and are influenced by modifiable and nonmodifiable factors, including interventions [4,6,[14], [15], [16]]. Here, we expand on these reviews by reporting the current knowledge regarding associations between specific HM micronutrients and child anthropometrics among healthy, term-born infants.

## Methods

This review is reported according to the PRISMA guidelines [17] and registered with PROSPERO (registration no. CRD42020187350) [18]. A team of 8 reviewers (SMR, JMM, DC, MG, KKS, SM, RR, and MB) participated in abstract and full-text screening, quality assessment, and data extraction.

### Search strategy and screening

A health sciences librarian (CM) developed and tested the search strategy in consultation with the review team. We used a combination of controlled vocabulary and keywords to create search concepts for HM, growth and development, macronutrients, micronutrients, and bioactive components. We also included an infant search filter adapted from the Pediatric Search Filter from the Cochrane Childhood Group to limit ≤24 mo of age. The search was peer-reviewed by another health sciences librarian using the Peer Review of Electronic Search Strategies method [19]. The original search strategy was created in MEDLINE (Ovid) and translated to other databases. The MEDLINE (Ovid) strategy is available in Appendix A. All other strategies are available upon request.

The following databases were searched in March 2020: MEDLINE (Ovid; MEDLINE All 1946–2020), Embase (Ovid; 1974–2020), the Cochrane Library (Wiley; CENTRAL and Cochrane Database of Systematic Reviews), Scopus (1970–2020), and Web of Science Core Collection (Clarivate, 1900–2020). To locate gray literature, we searched Agricola, PEN (Practice-based Evidence in Nutrition), OpenSIGLE, Google Advanced, and PROSPERO. These resources were selected to ensure the retrieval of materials relevant to nutrition, food science, and technology. Finally, reverse snowballing (using the reference list of a paper to identify additional papers) [20] was conducted on review articles identified with our search strategy. References published in English and after 1980 were included. The search was updated in March 2022, revisiting all the original databases and gray literature sources. The records were exported into Endnote version x9 (Clarivate Analytics), and duplicates were removed [21]. All records were screened in duplicate in Covidence (Veritas Health Innovation).

### Selection criteria

Search results were screened in duplicate. Any randomized controlled trial (RCT) or observational study was eligible for inclusion if it reported associations between HM components and infant anthropometrics. Data from RCTs were evaluated as observational studies because, in all cases, associations between HM composition and infant anthropometrics were secondary analyses. We required that studies reported on healthy, term, HM-fed infants (aged 0–24 mo). Healthy was defined as term birth (37 wk, 0 d of gestation or later) with no congenital or other morbidities and no admission in the neonatal intensive care unit, as described by the study authors. Studies that included preterm infants were excluded unless it was possible to extract data for the term infants separately. Although breastfeeding exclusivity was not an inclusion criterion, it was recorded when reported by the authors. Our main outcomes were: weight-for-age *z*-score (WAZ), length-for-age *z*-score (LAZ), weight-for-length *z*-score (WLZ), BMI or BMI-for-age *z*-score, and growth velocity. Reference populations used to calculate *z*-scores varied across studies, and some studies reported percentiles rather than *z*-scores. To simplify the synthesis of results, *z*-scores and percentiles were summarized together.

We also included articles that reported other infant anthropometrics, including but not limited to weight, length, rapid weight gain (as reported by study authors), total adiposity (%BF by DXA or skinfold thickness), body composition (FM, FFM, %FM by bioelectrical impedance spectroscopy or skinfold thickness), stunting, wasting, under- or overweight, and head circumference.

### Quality assessment

Articles were assessed for quality using a modified Newcastle-Ottawa scale [22] (Supplementary Table 1). Based on previous research [23] and in collaboration with multiple subject matter experts, we created a 17-point evaluation scale. We designated 8 points *for HM exposure assessment*, including HM collection and handling protocol (3 points), HM sample preparation (1 point), analytical method used to measure HM analyte (2 points), longitudinal HM sampling strategy (1 point), and accounting for infants’ HM intake (1 point); *5 points for confounders considered*, including infant diet (2 points), birth anthropometrics (1 point), baseline characteristics of mothers and infants (2 points); and *4 points for infant anthropometry outcome assessment,* including whether infant anthropometrics were measured by trained staff (1 point) using technical replicates (1 point), longitudinally (1 point), and with all infants measured within 1 wk of each other at each time point (1 point). Quality assessment for each article was conducted in duplicate, with conflicts addressed through consensus. Overall quality scores >13–17 were considered high, 7–13 moderate, and <7 low. Quality scores were also evaluated individually for exposure assessment (high: >6–8; moderate: 3–6; low: <3), confounders considered (high: >4–5; moderate: 3–4; low: <3), and outcome assessment (high: >3–4; moderate: 2–3; low: <2).

### Data extraction

Data extraction was conducted using a standardized form that was developed and piloted in collaboration with subject matter experts. Data extracted included: publication year, location, design, baseline characteristics of mothers and infants, HM sampling times, the timing of infant anthropometric measurements, whether HM micronutrient values were reported as concentrations or estimated daily intakes, outcomes, associations reported (correlations and unadjusted and adjusted β-estimates, as reported by study authors), and major confounders considered (via study design or statistical analyses) including maternal age, parity, maternal BMI (as defined by study authors), ethnicity, time postpartum, breastfeeding exclusivity, birth anthropometrics, infant age and sex, and any others reported. Study authors were contacted to request data in instances they were missing or presented in non-extractable formats. In 1 case, additional data were provided in a dissertation written in French. Translational services were acquired to interpret the results. Each article was extracted in duplicate, and conflicts were addressed through consensus.

### Analytical strategies

Data were summarized in tables as reported by study authors, and directional associations reported for HM concentrations were visualized in a heatmap with colors determined by synthesizing evidence across studies according to the presence and direction of associations. When the direction was discordant across studies, evidence was considered “mixed.” Some papers only reported associations between HM micronutrients and infant anthropometrics that were statistically significant; for these studies, we considered the unreported associations as “assumed no association.” Meta-analysis was not possible for any micronutrient due to heterogeneity in study designs, sampling times, and reporting practices. These limitations also prevented subgroup or sensitivity analyses that were planned on the following a priori criteria: study setting, birth weight, breastfeeding status, mode of HM delivery, gestational age, infant sex, and nutritional status of mothers. The full list of prespecified subgroups is available in the review protocol [18].

## Results

### Description of included studies

**FIGURE 1:**
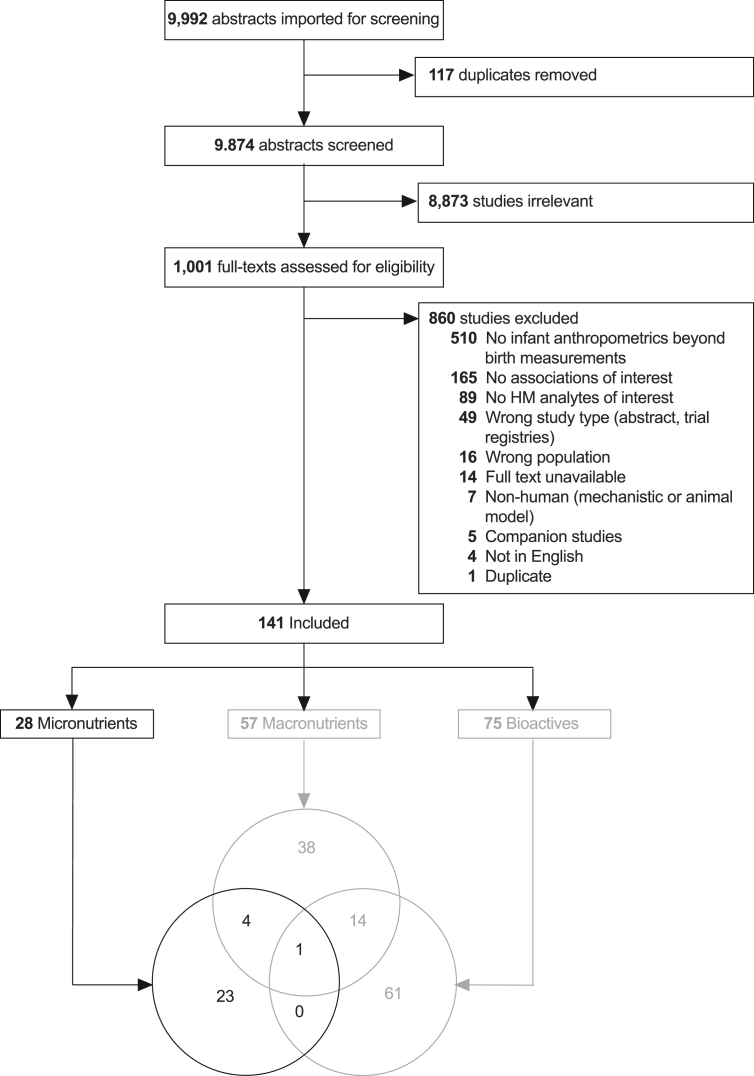
Systematic review of associations between human milk micronutrients and infant growth in the first 2 y: PRISMA flow diagram. Reasons for study exclusion were recorded in the order listed in the figure. Though some studies had >1 reason for exclusion, each study was only counted once (e.g., if a study reported no human milk analytes of interest and was not in English, it was recorded as the former). Micronutrient studies are reported in the current paper; Macronutrient and Non-nutritive Bioactive studies are reported separately.

In total, 9992 unique abstracts were identified, 1001 full texts were assessed, and 139 articles met inclusion criteria, of which 28 articles reported HM micronutrients [[24], [25], [26], [27], [28], [29], [30], [31], [32], [33], [34], [35], [36], [37], [38], [39], [40], [41], [42], [43], [44], [45], [46], [47], [48], [49], [50], [51]] (Figure 1). The main reasons for excluding articles were: no HM analytes of interest were reported (*n* = 89); no infant anthropometrics or only birth anthropometrics were reported (*n* = 512); or no associations between HM analytes and infant anthropometrics were reported (*n* = 165). Together, these 3 reasons accounted for 77% (766/1001) of the studies excluded during full-text screening.

Among the 28 included micronutrient articles (Table 1 and Supplementary Table 3), 8 (29%) were published before 1998 [27,29,31,35,38,39,41,45]; 19 (67%) were published in 2010 or later [25,26,28,30,[32], [33], [34],^36^,^37^,^40^,[42], [43], [44],[46], [47], [48], [49], [50], [51]]. These 28 articles represented 26 unique studies involving 2526 dyads; 17 studies were conducted in low- or middle-income countries (according to World Bank criteria) [[24], [25], [26],^30^,[32], [33], [34],[36], [37], [38], [39],[41], [42], [43], [44],^46^,^48^,^49^]. Five studies were conducted as diet interventions or maternal micronutrient trials [24,31,36,44,49].

**TABLE 1:** Detailed characteristics and results of included studies reporting on human milk micronutrients and infant anthropometrics (organized alphabetically by first author). An alternative version organized by nutrient is available in Supplementary Table 3

Authors, publication year, country (income setting)	Design and participants	Milk sampling time(s), analytes and units	Anthropometric outcome assessment time(s), measures, and standards	Associations^2^	Major confounders considered
Bilston-John et al. [51] (2021), Australia (HIC)	Cohort 83	1, 2, 3, 4, 5, 6, 9, 12 mo	3, 6 mo	(No) Association for any micro or macro minerals and anthropometrics	None
		Ca, Na, K, P, Mg, I, Se, Cu, Fe, Mo, Mn, Zn (concentration and estimated intake)	Weight, length, HC, LAZ, HCFAZ, WAZ (WHO standards)		

Butte et al. [27] (1987), United States (HIC)	Longitudinal 45	1, 2, 3, 4 mo	1, 2, 3, 4 mo	(+) Associations for Ca, P, Mg, and K and weight and WFA (+) Associations for Ca and P and growth rate	None
		Ca, P, Mg, Zn, Na, K, Fe, Cu (estimated intake)	Weight, WFA percentile, growth rate (g/d) (NCHS growth curves)	(No) Assumed associations for all other combinations of milk analytes and anthropometrics	

Casterline et al. [39] (1997), Guatemala (LMIC)	Cross-sectional 113	3 mo Vitamin B12 (concentration)	3 mo WAZ, HAZ, WHZ	(No) Associations for vitamin B12 and WAZ, HAZ, WHZ	None

Cissé et al. [24] (2002)^1^, Senegal (LMIC)	Longitudinal 133; 41 mothers supplemented with millet (high protein); 35 supplemented with maize (high CHO); 57 unsupplemented	14 d K, Mg, Ca, Zn, Na (concentration)	3 mo WFL, LFA (percentiles) (NCHS growth curves)	(+) Association for K, Mg, Ca, and WFL (all groups)	None
				(+) Association for K and Ca and LFA (all groups)	
				(+) Association for Zn and Na and WFL (supplemented groups only)	

Dorea [41] (1993), Brazil (LMIC)	Longitudinal 8	0, 1, 2, 3, 4, 5, 6 mo Zn (concentration)	Ponderal growth, linear growth (WHO standards)	(+) Associations for Zn and ponderal and linear growth in first 6 mo	Total nitrogen and fat

Doneray et al. [32] (2017), Turkey (LMIC)	Longitudinal 37	8–12 d, 25–30 d, 2 collections each (foremilk and hindmilk)	8–12 d, 25–30 d weight	(−) Associations for Zn (hindmilk) and weight in early lactation	None
		Zn (concentration)		(+) Associations for Zn (hindmilk) and weight in late lactation	

Dumrongwongsiri et al. [48] (2022), Thailand (LMIC)	Cohort 120 (64 and 56 analyzed)	2, 4 mo Zn, Fe (concentration and estimated intake)	2, 4 mo Weight, height, weight gain, length gain, WAZ, LAZ, WFLZ, WLZ (WHO standards)	(+) Association for Zn and weight gain (birth - 4 mo) and WLZ	Age, anthropometric measurement, and dietary intakes
				(No) Association for Zn and length gain or LAZ	
				(No) Association for Fe and weight gain, length gain, WLZ or LAZ	

Ellsworth et al. [28] (2020), United States (HIC)	Longitudinal 57 (35 analyzed)	2 wk	2 wk, 2, 6, 12 mo	(+) Associations for I and WFA, WLZ	Maternal: BMI (assumed to be prepregnancy), the interaction of maternal BMI and time; Infant: birth anthropometric *z*-score, gender
		I (concentration)	WAZ, LAZ, WLZ (WHO standards)	(No) Association for I and LAZ	

Han et al. [43] (2009)^1^, Korea (LMIC)	Longitudinal 20	1, 2, 4, 5, 6 mo	1, 2, 4, 5, 6, 12 mo	(No) Associations for folate and weight or length	None
		Folate (concentration)	Weight, length		

Han et al. [34] (2011)^1^, Korea (LMIC)	Longitudinal 20	1, 2, 4, 5,6 mo Zn, Fe, Cu (estimated intake)	1, 2, 4, 5, 6, 12 mo Weight, length	(−) Association for Zn and weight but not length at 1 mo	None
				(−) Association for Cu and weight but not length at 1, 5 mo	
				(No) Associations for Fe and weight, length	

Han et al. [47] (2021), China (LMIC)	Cross-sectional 450 (264 analyzed)	42 d Se (concentration)	42 d	(−) Association for Se and WLZ (No) Association for Se and LAZ or WAZ	Daily dietary intake of total energy and 3 macronutrients, household income, age, parity, *z*-scores at birth
			Stunting (WHO standard)		
			LAZ < −2 (stunting)		
			WAZ < −2 (underweight)		
			WLZ = −2 (wasting)		
			WLZ = 2 (overweight)		
			WAZ > 2 (overweight)		
			WLZ > 3 (obesity)		

Hussein et al. [38] (1987), Egypt (LMIC)	Cross-sectional 14 healthy	5 to 53 wk	5 to 53 wk	(+) Associations for retinol and BMI	None
		Vitamin A (concentration)	Arc, the sin of the square root of (weight/height^2^-A)		

Jarjou et al. [30] (2012), the Gambia (LMIC)	Longitudinal 30	3, 12 mo	3, 12 mo	(+) Associations for Ca and weight and length at 3 and 12 mo, but no associations with BMC, BW, or BMD	Infant sex; BMC model adjusted for bone width, weight, and length to give a size-adjusted BMC
		Ca (concentrations and intakes)	Weight, length, BMC, bone width, and BMD		

Kang-Yoon et al. [31] (1992), United States (HIC)	Longitudinal 20; 7 mothers supplemented with 2 mg pyridoxine (PN-HCl); 7 mothers supplemented with 2 mg PN-HCl + infants supplemented with 0.4 mg PN-HCl; 6 mothers supplemented with 27 mg PN-HCl	7 d (foremilk)	7, 14, 28 d	(+) Associations for vitamin B6 and weight gain before adjustment for covariates	Birth weight, plasma pyridoxal-5'-phosphate
		Vitamin B6 (estimated intake)	Weight gain		

Krebs et al. [29] (1994), United States (HIC)	Longitudinal 71	2 wk, 1, 2, 3, 4, 5, 6, 7 mo; Females followed through 9 mo	2 wk, 1, 2, 3, 4, 5, 6, 7 mo; Females followed through 9 mo	(+) Associations for Zn and WAZ, WLZ, and HCAZ, only at distinct times (see Supplementary Table 1)	Milk volume intake
		Zn (estimated intake)	WAZ, WLZ, HCAZ (NCHS growth curves)		

Li et al. [25] (2016), Guatemala (LMIC)	Cross-sectional 234; 56 early milk samples (5–17 d); 75 transitional milk (18–46 d); 103 established milk (4–6 mo)	Cross-sectional, 3 groups: Early (5–17 d) Transitional (28–46 d) Established (4–6 mo) Multivariable analyses of Ca, Cu, Fe, Mg, Mn, K, Rb, Se, Na, Sr, Zn (concentration)	Same as milk sampling, cross-sectional WAZ, LAZ, HCAZ (WHO standards)	(+) Associations for multiple micronutrients (PC components) and WAZ, LAZ, and HCAZ during early and established but not transitional lactation	Infant sex, maternal height, shipment
				*Early lactation:* PC1 (intakes of Ca, P, Mg, Rb, Sr) associated with WAZ, LAZ, HCAZ; PC2 (intakes of Cu, Na, Se, Zn) associated with WAZ	
				*Transitional lactation:* None of the 3 PCs were significant in any of the regression models.	
				*Established lactation:* PC1 (Cu, K, Mg, Na, Rb, Sr) associated with WAZ, LAZ, HCAZ; PC3 (Cu, Se, Zn) associated with WAZ	

Li et al. [26] (2019), Guatemala (LMIC)	Longitudinal 114; 80 - without SCM (non-SCM) during early lactation (2–46 d); 34 - SCM in early lactation; 103 - non-SCM in established lactation (4–6 mo); 11 - SCM in established lactation (4–6 mo)	Early (2–46 d) and established lactation (4–6 mo) Ca, Cu, Fe, Mg, Mn, P, Se, K, Zn (concentration)	Early (2–46 d) and established lactation (4–6 mo) WAZ, LAZ, WLZ, HCAZ, at early lactation; growth velocity for weight, length, and HC between early and established lactation (WHO standards)	(+) Associations for Mn and WAZ, but not LAZ, WLZ, HCAZ, or growth velocity	Early lactation models: somatic cell count, milk cytokine concentrations, estimated milk intake Growth trajectory models additionally include: infant sex, early length or HC, maternal weight, height, and mixed feeding (yes/no)
				(−) Associations for Zn and HCAZ, but not WAZ, LAZ, WLZ, or growth velocity	
				(−) Associations for Mg and linear growth velocity, but not WAZ, LAZ, WLZ, or HCAZ	
				(No) Assumed associations for Ca, Cu, Fe, Mg, P, Se, K and WAZ, LAZ, WLZ, HCAZ, or growth velocity (weight, length, HC)	

Mahdavi et al. [33] (2010), Iran (LMIC)	Cross-sectional 182	90–120 d	90–120 d	(+) Associations for Zn and WAZ, but only among infants whose mothers with high values of milk Zn (No) Associations for Zn and HAZ	Rural vs. urban setting; maternal BMI, height, dietary EI; infant birth weight
		Fe, Zn, Cu (concentration)	WAZ, HAZ	(No) Associations for Cu, Fe, and WAZ or HAZ	

Minato et al. [40] (2019), Japan (HIC)	Longitudinal	1, 3 mo Ca, P (concentration)	1, 3 mo weight, length	(No) Associations for Ca, P, and weight or length at 1 or 3 mo	None
	56 (1 mo)				
	42 (3 mo)				

Motoyama et al. [50] (2021), Japan (HIC)	Cohort 129 (79 analyzed)	1, 3 mo Cr, Mn, Fe, Cu, Zn, Se (concentration)	1, 3 mo weight, height, HC	(No) Association for any trace elements and length, weight, or HC	None

Nazeri et al. [42] (2020), Iran (LMIC)	Longitudinal 94	3–5 d	6 mo	(+) Associations for I and WFLZ	Maternal age, educational level, prepregnancy maternal BMI, parity, type of feeding at 6 mo, birth anthropometric measurements; IGF-I, adiponectin, leptin
		I (concentration)	WAZ, LAZ, WFLZ, HCAZ (WHO standards)	Associations differ based on the presence of IGF-I, adiponectin, or leptin (No) Associations for I and WAZ, LAZ, HCAZ	

Nikniaz et al. [44] (2019), Iran (LMIC)	Cross-sectional 57; 30 in the synbiotic group; 27 in the placebo group	Unclear; before intervention	Unclear; before intervention	(+) Associations for high HM Se and WAZ, HAZ	None
		Selenium (concentration)	WAZ, HAZ		

Palmer et al. [36] (2016)^1^, Zambia (LMIC)	Longitudinal 149; 49 (white maize + placebo); 50 (orange maize + placebo); 50 (white maize + vitamin A)	4–12 mo (once pre- and post-intervention)	4–12 mo (once pre- and post-intervention) Weight, length	(No) Associations for vitamin A and provitamin A and weight, length (pre- or post-intervention)	None
		Vitamin A (Retinol), provitamin A (beta-carotene) (concentration)			

Salmenpera et al. [35] (1994), Finland (HIC)	Longitudinal 200	2, 4, 6, 7.5 mo	2, 4, 6, 7.5 mo	(+) Associations for Zn and weight velocities	None
		Zn (intake and concentration)	Weight, length, weight- and length-squared index, subscapular and bicep skinfold thickness	(No) Associations for Zn intake nor milk Zn concentration and weight or length-squared index, skinfold thickness	

Samuel et al. [37] (2014), India (LMIC)	Longitudinal 58 (50 followed to 6 mo)	1, 3, 6 mo	1, 3, 6 mo	(No) Associations for Zn and weight and length gain	Non-breast milk water intake, infant age, gender, weight, and length at birth and mo 3
		Zn (estimated intake)	Weight, length		

Sievers et al. [45] (1992)^1^, Germany (HIC)	Longitudinal 10	45 collection times during the first 17 wk	45 sampling times during the first 17 wk	(−) Associations for Zn and weight	non-breast milk water intake, infant age, gender, weight, and length at birth and mo 3
		Zn (concentration)	Weight		

Umeta et al. [46] (2003), Ethiopia (LMIC)	Cross-sectional 305 (253 analyzed)	1 collection between 5 and 11 mo	1 sample between 5 and 11 mo	(+) Associations for low milk Zn and stunting	Infant age
		Zn, Ca, Cu (concentration)	Stunting (NCHS)	(No) Associations among milk Ca, Cu, and stunting	

Young et al. [49] (2021), Guatemala, India, Pakistan (LMIC)	RCT 200	2 wk	1, 3, 6 mo	(No) Associations for assessing analytes and anthropometry	Maternal preconception BMI category, site, study arm
		Thiamin, vitamins B2, B3, B6, B12, pantothenic acid, biotin, choline (concentrations)	LAZ-slope, WAZ-slope, WLZ-slope		

Ca, calcium; CHO, carbohydrate; Cu, copper; Fe, iron; HAZ, height-for-age *z*-score; HC, head circumference; HCAZ, head circumference *z*-score; HCFAZ, head circumference-for-age z-score; HIC, high-income country; HM, human milk; I, iodine; K, potassium; LAZ, length-for-age *z*-score; LFA, length-for-age; LMIC, low and middle-income country; Mg, magnesium; Mn, manganese; Mo, Molybdenum; Na, sodium; P, phosphorus; PC, principal component; PN-HCl, pyridoxine; Rb, rubidium; RCT, randomized controlled trial; SCM, subclinical mastitis; Se, selenium; Sr, strontium; WAZ, weight-for-age *z*-score; WFA, weight-for-age; WFL, weight-for-length; WFLZ, weight-for-length z-score; WHZ, weight-for-height *z*-score; WLZ, weight-for-length *z*-score; Zn, zinc.

1 Indicates data were provided by the study author and do not appear in the referenced publication.

2 No (assumed) associations = unreported associations assumed to be no association.

Following weight and length measures, the most common outcomes reported were height- or length-for-age percentiles or *z*-scores (LAZ; 13 articles), weight (12 articles), weight-for-age percentiles or *z*-scores (WAZ; 11 articles), length (9 articles); and weight-for-length percentile or *z*-score (7 articles) (Table 1 and Supplementary Table 3). No study reported BMI or BMI-for-age *z*-score, rapid weight gain, total adiposity, MUAC, wasting, or under or overweight as outcomes.

### Study quality

**FIGURE 2:**
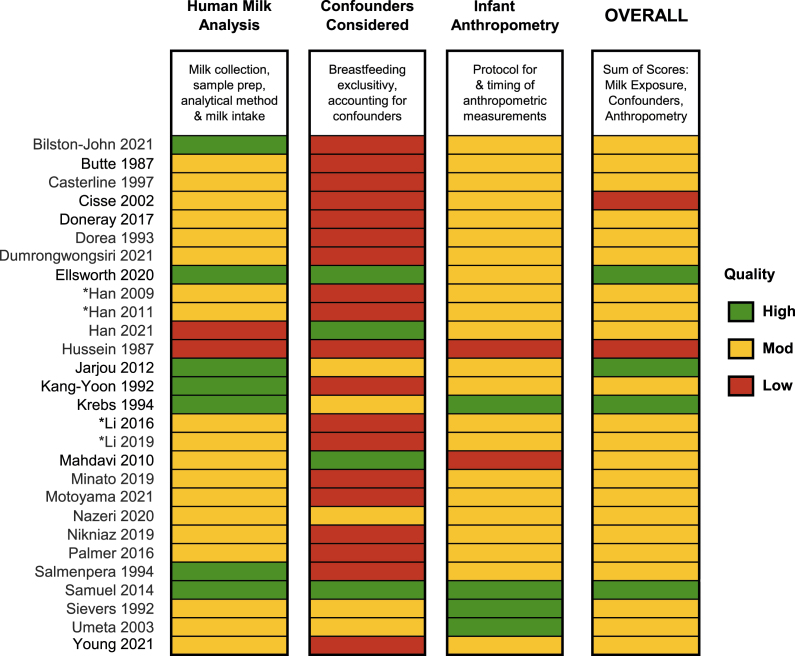
Association of human milk micronutrients and infant growth in the first 2 y: Summary of quality assessments of included articles. Asterisks denote companion articles from the same study. Quality scores are awarded based on the number of points assigned according to the criteria in Supplementary Table 1. Detailed numeric scores are presented in Supplementary Table 2.

Most studies (22/28; 79%) were of moderate quality (Figure 2 and Supplementary Table 2). Few (4/28; 14%) met our criteria for high overall quality. The most common limitation was a lack of consideration for relevant confounders (via study design or statistical analysis), such as maternal BMI, time postpartum, birth weight, and infant sex. Several studies also lacked details about complementary feeding or formula supplementation, even when follow-ups extended beyond 6 mo postpartum. Only 7 studies (25%) had high quality scores for exposure assessment [[28], [29], [30], [31],^35^,^37^]; the remainder received moderate or low-quality scores largely because of poor HM collection and/or reporting practices and lack of accuracy and precision estimates related to analytical methods used to measure HM micronutrients.

### Timing of HM collection and outcome measurements

**FIGURE 3:**
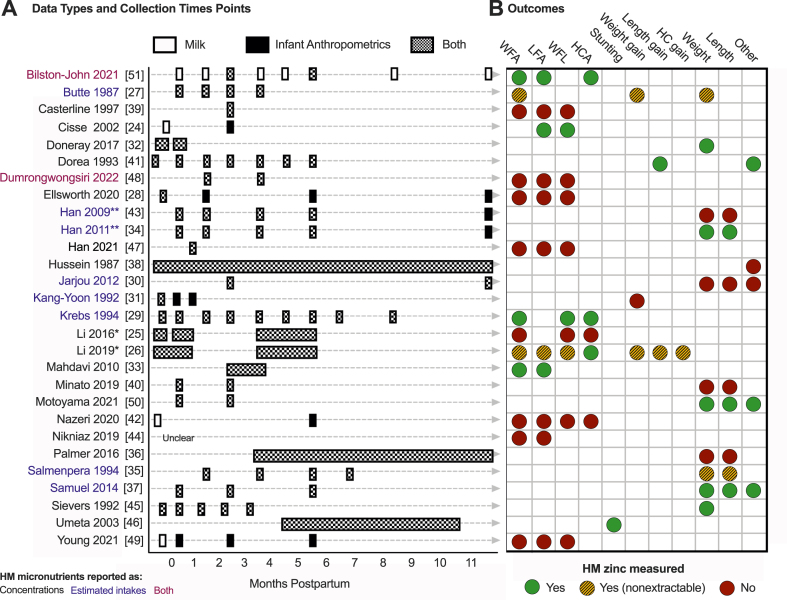
Association of human milk (HM) micronutrients and infant growth in the first 2 y: Summary of data collection and study time points (A) and anthropometric outcome measurements (B) for all included articles, highlighting zinc as an example. (A) Text color reflects that studies reported HM micronutrients as either estimated intakes (blue text) or concentrations (black text). Asterisks denote companion articles from the same study. The width of rectangles reflects the time postpartum measurements were taken. Narrow rectangles reflect that all measurements occurred approximately at the same time postpartum for all dyads. Wide rectangles reflect that the timing of measurements varied across dyads. (B) Zinc is highlighted here because it had the most evidence available, but even so, data were too sparse to conduct a meta-analysis. Whether HM zinc was measured and data were extractable is denoted as follows: yes (green circle); yes but not extractable (striped yellow circle); no (red circle).

Of the 28 publications, 7 were cross-sectional, and 21 were longitudinal (Table 1 and Supplementary Table 3). Among the 21 longitudinal investigations, 17 sampled HM in the first 6 mo only [24,[26], [27], [28],^31^,^32^,^34^,^37^,[39], [40], [41], [42], [43],^45^]; 3 also sampled HM between 6 and 12 mo [29,30,35] (Table 1, Supplementary Table 3, and Figure 3). Most studies measured HM analytes and infant anthropometrics concurrently [[25], [26], [27],^29^,^30^,[32], [33], [34], [35], [36], [37], [38], [39], [40], [41],^43^,^45^,^46^]. The median duration of follow-up for infant anthropometric measurements was 6 mo. The heterogeneity of sampling times, infant anthropometrics measured, and reporting practices across studies prevented meta-analyses, even among analytes for which there was the most data [e.g., Zn and calcium (Ca)] (Table 1 and Figure 3).

### Heterogeneity in reporting of results

There was substantial heterogeneity in how results were reported across studies. Some papers analyzed several HM analytes but only reported associations with infant anthropometrics that were statistically significant. For example, Butte et al. [27] investigated associations between estimated infant intakes of several HM micronutrients (Ca, phosphorous, magnesium [Mg], Zn, sodium [Na], potassium [K], Fe, and copper [Cu]) and infant weight, weight-for-age percentile, and growth rate (g/d) at 1, 2, 3, and 4 mo. However, results were only reported for Ca, Mg, K, and phosphorous for a subset of comparisons (Supplementary Table 3). In such cases, we assumed that the relationships not reported were not statistically significant (Figure 3B). Other studies explicitly stated that certain correlations were or were not statistically significant but did not provide extractable effect estimates [35,40,51].

### Estimated intake compared with concentration of micronutrients in HM

**FIGURE 4:**
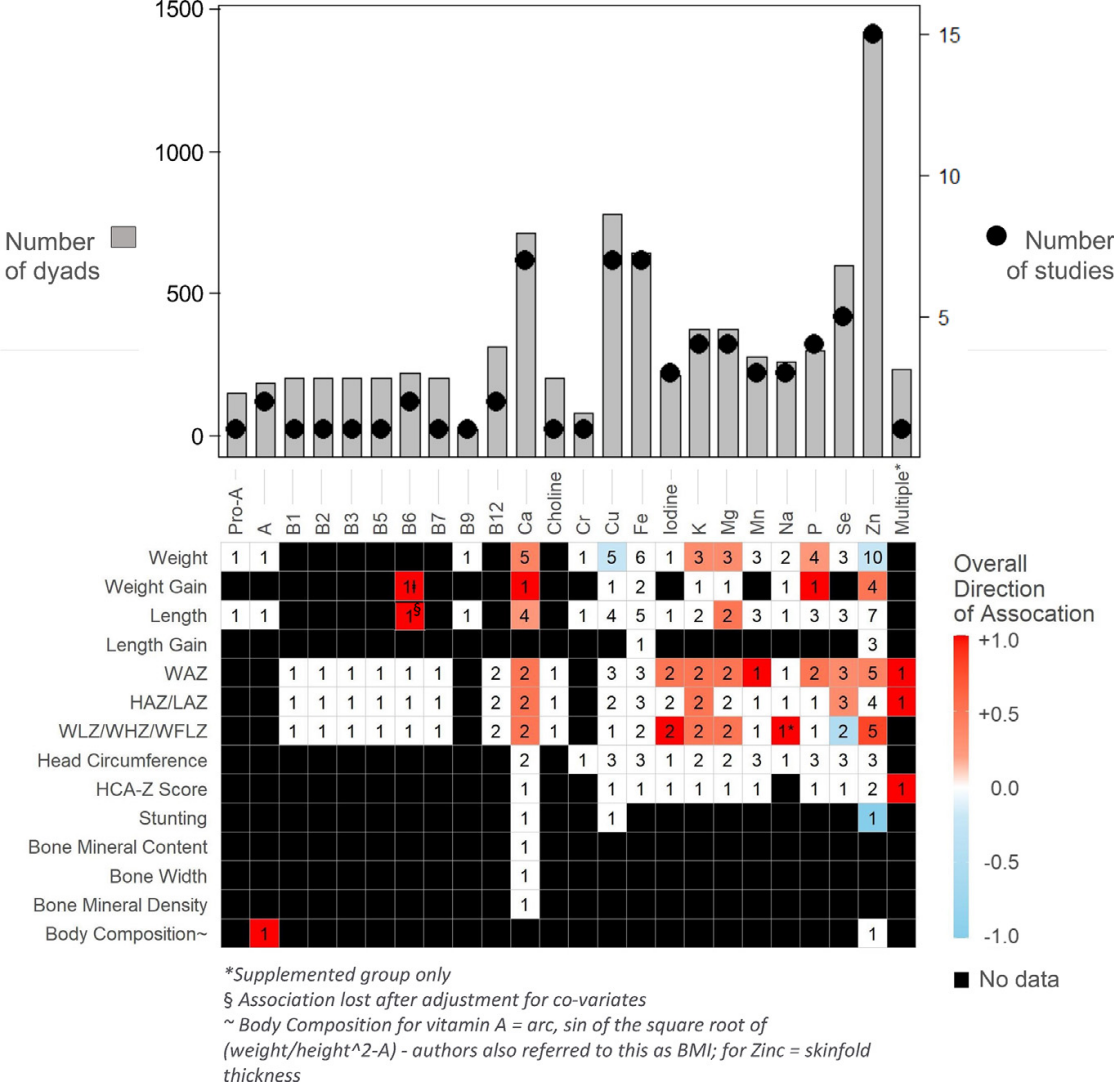
Association of human milk (HM) micronutrients and infant growth in the first 2 y: Summary results of all included articles. Associations between HM micronutrients and infant anthropometrics reflect results as reported by study authors (e.g., using HM concentrations or estimated intakes as the predictor variable, see Table 1). Red squares indicate positive associations, blue squares indicate an inverse association, white squares indicate no pooled association and black squares indicate association was not examined in any studies. The number in each square indicates the number of studies examining the association. Breastfeeding exclusivity was difficult to determine in most studies; thus, results summarize data from all included studies and may include infants with varying diets (see Supplementary Table 2). Multiple∗ indicates multiple micronutrients analyzed in multivariate models. B6, vitamin B-6; B12, vitamin B-12; HAZ, height-for-age *z*-score; HCA-Z, head circumference-for-age *z*-score; LAZ, length-for-age *z*-score; Pro-A, provitamin A/*β*-carotene; WAZ, weight-for-age *z*-score; WFLZ, weight-for-length z-score; WHZ, weight-for-height *z*-score; WLZ, weight-for-length *z*-score; ∗Supplemented group only; ± Association lost after adjustment for covariates; ∼ Body Composition for vitamin A = arc, the sin of the square root of (weight/height^2^-A) - authors also referred to this as BMI; for zinc = skinfold thickness.

Only 10 (35%) articles reported estimated intakes of HM micronutrients (Figure 3) [27,[29], [30], [31],^34^,^35^,^37^,^43^,^51^] [28,[30], [31], [32],^35^,^36^,^38^,^44^], with the remainder simply reporting HM micronutrient concentrations. Evidence suggests that the *intake* of HM nutrients is a better predictor of infant growth than nutrient concentrations in HM. For example, Jarjou et al. [30] (2012) used a stable isotope tracer in 30 mother-infant dyads in the Gambia to measure the total daily HM volume consumed alongside their analysis of HM micronutrient concentrations. They found that HM Ca intake (HM volume consumed x HM Ca concentration) positively predicted weight at 3 mo (*R*^*2*^ = 39%; *P* = 0.0002). However, when HM volume consumed and HM Ca concentration were included as independent variables in multiple regression models rather than combined as HM Ca intake, HM volume significantly predicted weight (*R*^*2*^ = 46%; *P* = 0.0001), whereas HM Ca concentration did not (*P* > 0.1) [30] (Table 1 and Figure 4).

### Vitamin A and provitamin A

Two studies (164 dyads) were included for vitamin A [36,38] (Table 1 and Figure 4). A cross-sectional study conducted in Egypt included 14 dyads between 5 and 53 wk postpartum and reported a positive correlation between HM retinol (vitamin A) concentrations and infant body composition [defined as the arc sign of the square root of (weight/height^2^-A); *r* = 0.57; *P* < 0.01] [38] (Table 1 and Figure 4). A 30-d pre/post dietary intervention conducted in Zambia included 149 dyads; mothers in the intervention group were supplemented with vitamin A. There was no correlation between HM retinol or *β*-carotene (provitamin A) concentrations and infant weight or length [36] (Table 1 and Figure 4).

### B vitamins

Four studies addressed B vitamins [31,39,43,49]. A micronutrient trial conducted in the United States reported that maternal vitamin B-6 intake was directly correlated with infant vitamin B-6 intake (Pearson *r* = 0.99; *P* < 0.0001) and infant vitamin B-6 status (measured as plasma pyridoxal-5’-phosphate [PLP], pyridoxal [PL], and PL/PLP ratio; Pearson *r*: 0.72–0.93; all *P* values *<* 0.006). Simple linear regression analysis revealed that infant vitamin B-6 intake from HM at 7 d was associated with weight gain from 7 to 28 d (*β* = 0.0004 ± 0.0001 weight *z*-score per μg of vitamin B-6/d; *P* = 0.01; *R*^*2*^ = 0.34; 20 dyads). The combination of infant vitamin B-6 intake, birth weight, PLP, and PL/PLP ratio in multiple regression accounted for 75% variation in weight changes observed from 7 to 28 d; most of the variation was associated with plasma PLP and birth weight [31] (Table 1 and Figure 4). Although plasma PLP is the most commonly used indicator of vitamin B-6 status, this measure can be confounded by inflammation and alkaline phosphatase activity and may not be a good indicator of estimated vitamin B-6 intake [52]. These findings suggest that infant vitamin B-6 status is a stronger predictor of weight gain than vitamin B-6 intake per se, which is a reasonable conclusion given that vitamin B-6 is water soluble, absorbed by passive diffusion, and thus influenced by status [48].

The Women’s First Trial conducted in Guatemala, India, and Pakistan [49] examined the impact of a lipid-based nutrient supplement on 8 vitamin B concentrations (thiamin, vitamins B-2, B-3, B-6, B1-2, pantothenic acid, and biotin) in HM. In this trial, supplementation had no meaningful impact on vitamin B concentrations in HM, and there were no associations between B vitamin concentrations and infant growth outcomes (LAZ, WAZ, and WLZ).

A cross-sectional study in Guatemala reported no correlations between HM vitamin B-12 concentrations and infant WAZ, height-for-age *z*-score (HAZ), or weight-for-height *z*-score at 3 mo [39] (Table 1 and Figure 4). A prospective longitudinal study in South Korea reported no associations between HM folate concentrations and infant weight or length from 1 to 6 mo [43] (Table 1 and Figure 4).

### Calcium

Six longitudinal studies (5 observational and 1 dietary intervention; 461 dyads) [24,26,27,30,40,51] and 1 cross-sectional study (253 dyads) [46]were included for Ca (Table 1 and Figure 4). One study assessed HM Ca concentrations and head circumference, finding no associations in the first 6 mo (114 dyads, Guatemala) [26] (Table 1 and Figure 4).

HM Ca intake, but not overall HM Ca concentration, appeared to be an important predictor of infant weight in early infancy in 1 study [27]. Among 45 dyads in the United States, HM Ca intake was positively correlated with infant weight gain (g/d) and weight-for-age at months 1, 2, and 4 [27]. Two additional studies reported positive associations between HM Ca intake and infant weight (75 dyads, 30 from the Gambia and 45 from the United States). In contrast, HM Ca concentration was not associated with infant weight at 1 or 3 mo [40] (56 dyads, Japan), and no significant associations were reported between HM Ca concentrations between 2 and 46 d and weight gain between 4 and 6 mo (114 dyads, Guatemala) [26] or among 83 dyads in Australia [51].

HM Ca intake appeared to be an important predictor of infant length from 6 to 12 mo in settings where maternal and infant undernutrition is common (there were no studies examining this association in high-resource settings). Among 30 dyads in the Gambia, HM Ca intake was strongly predictive of infant length at 12 mo (*R*^*2*^ = 32%; *P* = 0.001) but not at 3 mo (*R*^*2*^ = 13%; *P* = 0.056) [30] (Table 1). Moreover, HM Ca concentrations of mothers whose infants were stunted tended to be lower than those of mothers whose infants were not stunted [*β* = −1.3 mmol/L (95% CI: −2.7, −0.2); *P* = 0.08; between 5 and 11 mo] among 253 dyads in Ethiopia [46] (Table 1).

### K and Mg

Four longitudinal studies evaluated HM K and Mg concentrations and infant anthropometrics (381 dyads) [26,27,40,51] (Table 1 and Figure 4). No associations were reported for infant head circumference in the first 6 mo [26,27,51] (159 dyads) (Table 1).

Intakes of HM K and Mg intakes were independently correlated with infant weight in the first 2 mo, but not at months 3 and 4 [27] (45 dyads, United States) nor with overall weight gain in the first 6 mo [26,27] (159 dyads) (Table 1). Results were also mixed for weight-for-age [26,27] (159 dyads) and weight-for-length [24,26] (247 dyads).

The association between HM K concentration and length-related outcomes is unclear. There were positive correlations between HM K intake at 14 d and length-for-age at 3 mo among 133 dyads in Senegal [24]; however, there were no significant associations between HM K intake and length-related outcomes in the first 6 mo in the single study that adjusted for major confounders [26] (114 dyads, Guatemala) (Table 1 and Figure 4). In this study, HM Mg intake appeared to be negatively correlated with length gain but not HAZ at 2–46 d [26], although there was a negative correlation between HM Mg intake at 2–46 d and length gain from 2–46 d to 4–6 mo (adjusted *β* = −0.218 cm/mg/d; *P* = 0.0008) (Table 1 and Supplementary Table 3). Similarly, a study in Australia examining HM K intake in 83 dyads did not find any associations between HM K concentrations and infant weight, length, or head circumference [51].

### Sodium

Three longitudinal studies assessed the relationship between HM Na and infant anthropometrics [24,27,51]. Cissé et al. [24] (2002) conducted a dietary intervention (maize or millet supplementation compared with control) among women in Senegal. At 14 d, there was a positive correlation between HM Na concentrations and weight-for-length percentiles, but only for the intervention groups (133 dyads) (Table 1 and Figure 4). In contrast, Butte et al. [27] (1987) and Bilston-John et al. [51] (2021) each conducted prospective longitudinal studies in high-income countries and found no association between estimated HM Na intake and infant weight, weight-for-age percentile, or growth rate (g/d) ([27]; 45 dyads) or infant weight, length or head circumference ([51]; 83 dyads) (Table 1 and Figure 4).

### Phosphorus

Four studies were included for HM phosphorus (298 dyads) [26,27,40,51] (Table 1 and Figure 4) [28,41]. There were no associations between HM phosphorus and length- or head circumference-related outcomes in the first 6 mo [27,28,51] (242 dyads). Results were mixed for weight-related outcomes. Minato et al. [40] reported no significant correlations between HM phosphorus concentrations and infant weight at 1 and 3 mo (56 dyads, Japan). In contrast, Butte et al. [27] (1987) reported that HM phosphorus intake was associated with infant weight at 1 and 2 mo (45 dyads, United States).

### Zinc

Fifteen articles were included for Zn [24,26,27,29,[32], [33], [34], [35],^37^,^41^,^45^,^46^,^48^,^50^]. This was the largest evidence base available for any single micronutrient, but even so, meta-analyses were not possible due to heterogeneity in reporting practices and outcomes measured (Table 1 and Figure 4). Overall, results were mixed but suggested that Zn intake from HM may influence both weight (particularly in early infancy and when mothers are well-nourished) and length (particularly in later infancy).

#### HM Zinc and weight

Thirteen articles investigated HM Zn and infant weight-related outcomes [24,26,27,29,[32], [33], [34], [35],^37^,^45^,^48^,^50^,^51^] (Table 1 and FIGURE 3, FIGURE 4), with generally mixed results. Five articles reported on infant WAZ [26,29,33,48] or percentiles [27] (Table 1 and Figure 4); 2 found positive associations, whereas 3 reported no associations. Specifically, there were no associations between HM Zn intake and weight-for-age percentiles in the first 4 mo among 45 dyads in the United States [27]. Similarly, there were no assumed associations between HM Zn concentrations and WAZ in early lactation (2–46 d; 114 dyads, Guatemala) [26]. Finally, Dumrongwongsiri et al. [48] (2022) did not find any associations between HM concentrations or daily intake of Zn and infant weight-for-age percentiles. However, this study did find a positive association between Zn intake and infant weight gain from birth to 4 mo of age (Table 1). In contrast, Mahdavi et al. [33] (2010) reported higher WAZ in infants whose mothers had HM Zn concentrations >2 mg/L at 90–120 d (*β* = 0.16 ± 0.09 WAZ/mg/L; *P* = 0.09, 182 dyads, Iran). Additionally, Krebs et al. [29] (1994) found that changes in WAZ from 5 to 7 mo were positively associated with the percentage of Zn obtained from HM at 5 mo postpartum (*P* = 0.003; 71 dyads followed longitudinally in the United States; Table 1).

The reason for differing results across studies remains unclear but may be related to overall maternal nutritional status since maternal diet influences HM protein concentrations [53], and higher HM protein concentration improves infant Zn absorption [54]. Thus, improving the maternal diet would increase HM protein concentrations, leading to improved HM Zn absorption and improved infant growth outcomes. This possibility is supported by results from a trial in Senegal [24], where a positive association was observed between HM Zn concentrations at 14 d and weight-for-length percentiles at 3 mo among women supplemented with millet (*R*^*2*^ = 0.18; *P* = 0.007; *n* = 41) or maize (*R*^*2*^ = 0.16; *P* = 0.01; *n* = 35), but not among unsupplemented women (*R*^*2*^ = 0.05; *P* = not significant; *n* = 57). In this trial, supplemented mothers had significantly higher HM protein concentrations (15 ± 3 g/L protein [millet], 15 ± 2 g/L protein [maize] compared with 12 ± 3 g/L protein (unsupplemented); *P* < 0.01). Li et al. [26] also found no associations between HM Zn concentrations and WLZ at 2–46 d among unsupplemented women (114 dyads, Guatemala).

#### HM Zinc and length

In rural Ethiopia, where chronic malnutrition is highly prevalent [55], mothers whose infants were stunted had lower HM Zn concentrations than mothers whose infants were not stunted (*β* = −1.1 μmol/L [95% CI: −2.1, −0.1]; *P* = 0.02; 235 dyads, cross-sectional study of infants aged 5–11 mo) (Table 1 and Figure 4) [46]. Time postpartum may be an important factor for length-related infant outcomes. Krebs et al. [29] sampled HM and measured infant anthropometrics several times between 2 wk and 9 mo but only found a positive association between HM Zn intake at 7 mo and WLZ between 7 and 9 mo (*P* = 0.020; 40–71 dyads, United States), suggesting that HM Zn intake may be particularly important for length-related outcomes in the second half of infancy. Consistent with this, 9 additional studies found no association between HM Zn intakes or concentration and other length-related outcomes in the first several months [24,26,[33], [34], [35],^37^,^48^,^50^,^51^].

#### HM Zinc and head circumference

HM Zn may also be important for head circumference in the second half of infancy. Krebs et al. [29] found a positive association between the percentage of Zn obtained from HM at 7 mo and head circumference-for-age *z*-score (HCAZ) at 7–9 mo (*P* = 0.020; 71 dyads, United States; Table 1). In contrast, Li et al. [26] (2016), Motoyama et al. [25] (2021), and Bilston-John et al. [51] (2021) found no assumed association between HM Zn concentrations and head circumference measures in the first 6 mo (Table 1). Although not tested in these studies, Zn absorption may differ from HM compared with complementary foods, and it is possible that total Zn consumed is important in reducing illnesses and infections, resulting in improved growth. Alternatively, Zn may indirectly affect growth outcomes by influencing immune function and appetite [56].

### Copper

Seven articles were included for Cu (776 dyads) [26,27,33,34,46,50,51] (Table 1 and Figure 4). There were no reported associations between HM Cu and any infant anthropometric outcomes in the first year, with 1 exception. Han et al. [34] reported negative associations between HM Cu intake and infant weight at 1 and 5 mo (20 dyads, Korea) (Table 1). However, analyses were not adjusted for important confounders such as birth anthropometrics and complementary feeding or formula supplementation.

### Iodine

Three articles were included for iodine (212 dyads) [28,42,51]. Nazeri et al. [42] and Ellsworth et al. [28] each reported positive associations between HM iodine concentrations in the first 2 wk and infant WLZ between 2 wk and 1 y. Specifically, Nazeri et al. [42] found that HM iodine concentrations at 3–5 d were associated with WLZ at 6 mo, after adjustment for HM hormones (IGF-I, adiponectin, and leptin), maternal BMI, parity, infant feeding at 6 mo, and birth anthropometrics (94 dyads, Iran). Similarly, Ellsworth et al. [28] reported that a higher HM iodine concentration at 2 wk was associated with a larger increase in infant WLZ change per month from 2 wk to 1 y (*β* = 0.00029 WLZ/ng/mL iodine; *P* = 0.021), after adjustment for maternal BMI and infant birth anthropometrics (35 dyads, United States). Conversely, Bilston-John et al. [51] found no associations between HM iodine (intake or concentration) and infant weight, length, or head circumference.

### Iron

Seven included studies reported no associations between HM Fe and infant anthropometric outcomes in the first year (361 dyads) [26,27,33,34,50,51] (Table 1 and Figure 4). These results were expected as their concentrations are generally low in HM, and infants rely on body stores during early infancy to meet their needs for these nutrients [4].

### Selenium

Five articles were included for selenium (Se) (597 dyads) [26,44,47,50,51], reporting discordant results for WAZ and LAZ. Li et al. [26] reported no significant associations between HM Se concentrations and WAZ or HAZ during early lactation (2–46 d; 114 dyads, Guatemala) (Table 1 and Figure 4). Similarly, Motoyama et al. [50] and Bilston-John et al. [51] reported no association between HM Se and infant weight, length, or head circumference. In contrast, Nikniaz et al. [44] reported that mean infant WAZ and HAZ were significantly higher for infants whose mothers’ HM Se concentration was >60 μg/L at baseline (57 dyads, Iran). Conversely, Han et al. [47] found that HM Se concentrations were inversely associated with infant WLZ. The reason for these discordant results is unclear because, for some studies, insufficient details were provided for study methods, infant age, sampling times, and statistical analyses.

### Manganese

Three articles reported HM manganese (Mn) concentrations and infant anthropometrics [26,50,51]. Among 114 dyads in Guatemala, HM Mn concentration was positively associated with WAZ during early lactation (2–46 d) (*β* = 0.211 WAZ/μg Mn/d; *P* = 0.021, adjusted for somatic cell count, HM cytokine concentrations, maternal weight, and complementary feeding) [26] (Table 1 and Figure 4). There were no reported associations between HM Mn concentration and LAZ, WLZ, or HCAZ during early lactation or with growth velocity between early and established lactation (4–6 mo). Two studies conducted in high-income settings (162 dyads, Australia and Japan) also failed to find any association between HM Mn and infant weight, length, or head circumference measures [50,51].

### Multivariate relationships between HM micronutrients and infant anthropometrics

Only 1 study applied statistical methods to account for multiple micronutrients simultaneously in relation to infant anthropometrics [25]. In this cross-sectional analysis of 234 dyads in Guatemala at 3 different stages of lactation (5–17 d; 18–46 d; 4–6 mo), Li et al. [25] used ordination analyses to report the combined associations of Ca, Cu, Fe, Mg, Mn, K, rubidium (Rb), Se, Na, strontium (Sr), and Zn concentrations and infant WAZ, LAZ, and HCAZ. There was a positive association between the first principal coordinates axis (PC1, intakes of Ca, K, Mg, Rb, Sr) and WAZ, LAZ, and HCAZ during early (5–17 d) and established lactation (4–6 mo), but not during transitional lactation (18–46 d). These models captured a 41.7% variation of WAZ during early lactation and 67.4% during established lactation. Notably, the authors [26] reported much smaller *R*^*2*^ values (≤10.4% variation captured) when they initially applied traditional methods to study individual nutrients, suggesting that multivariable approaches that combine HM analytes have more predictive power than can be achieved by analyzing them separately.

### Sensitivity analyses and publication bias

Because studies varied markedly by design, sampling times, and outcomes measured, it was not possible to conduct the planned sensitivity analyses or formally assess publication bias. However, as described above for Zn, we found suggestive evidence that relationships between HM micronutrients and infant anthropometrics may depend on overall maternal nutrition status, which varies by country income level. Among the studies included, data were sometimes missing for nonsignificant values, suggesting the possibility of publication bias.

## Discussion

### Key findings

This systematic review reveals that little is known about how individual HM micronutrients influence infant and young child anthropometrics. Data were sparse for many micronutrients and despite the WHO recommendation to breastfeed for ≤2 y or longer [57], most evidence was limited to the first 6 mo postpartum. Five studies comprising 454 dyads [24,26,27,50,51] represented most of the information known about K, Mg, Ca, Na, Mn, Se, and phosphorus. No eligible studies were identified for fluoride, cobalt, chloride, sulfur, riboflavin, chromium, molybdenum, cobalt, and vitamins C, D, E, and K. Even where multiple studies were identified, meta-analyses were not possible because studies varied markedly in sampling times, anthropometrics measured, and reporting practices.

Notably, 165 publications *could have* contributed to this literature because they reported on HM micronutrients and child anthropometrics but not their associations (typically, these studies were focused on associations between maternal characteristics and HM composition). Accordingly, more is known about the determinants of HM composition than its subsequent impact on infant growth. These existing data could potentially be leveraged to improve knowledge about the associations between HM micronutrients and child anthropometrics.

Overall, we observed that current evidence suggests a positive correlation between infant growth and HM concentrations of iodine, Mn, Ca, and Zn, though these relationships remain largely unclear due to sparse data, small sample sizes, and methodological limitations of existing studies.

### A clear need for high-quality research

Many of the included studies suffered from poor quality. Only 6 studies (21%) had high-quality scores for “HM exposure assessment” [[28], [29], [30], [31],^35^,^37^], indicating a clear need for harmonized HM collection and reporting protocols [23,58]. Many studies lacked adequate details about HM collection, which can strongly impact nutrients that are fat-soluble, light-sensitive, or quickly metabolized [59]. For instance, total fat and fat-soluble vitamin concentrations vary considerably between foremilk and hindmilk and throughout the day [6], and iodine concentrations vary considerably with supplement use and meals [60,61]. Other studies failed to report key metrics related to the validation of HM assays (e.g., accuracy and precision), and very few attempted to estimate the *intake* of HM micronutrients - which requires a measurement of HM consumption in addition to micronutrient concentration.

Most studies (75%) had low-quality scores for “confounders considered” because they failed to account for key confounders such as birth weight, infant sex, maternal BMI, time postpartum, and infant diet (e.g., formula supplementation or complementary feeding). Also, few studies investigated the possibility of effect modification by factors such as chronic infectious disease, parasites, maternal nutritional status, or food insecurity.

### Absence of evidence is not evidence of absence

The associations between HM micronutrients and infant growth remain unclear largely because of a lack of evidence stemming from methodological limitations of existing literature (as discussed above) and a lack of investigation. This general lack of evidence makes it difficult to draw conclusions from existing literature. For instance, studies included in this review found no correlation between vitamin B12 in HM and infant anthropometrics; yet, it is well-known that infants born to mothers with poor vitamin B12 status have a relatively high incidence of poor growth [62]. These seemingly contradictory results are likely explained by the fact that existing evidence on vitamin B12 deficiency in infants primarily comes from case studies [62], which were excluded from this review. Additionally, Ca is important for bone mineralization, and bone Ca accumulation is highest during infancy [63], so a positive correlation would be expected between HM Ca intake and infant linear growth. Yet, evidence was mixed on the association of HM Ca with these outcomes, and 1 study demonstrated that correlations between HM Ca intake and infant growth were driven by overall HM intake rather than by Ca per se. This also highlights the complexity of exposure assessments relevant to anthropometric outcomes, including interventions such as antibiotic therapy. Clearly, more foundational research is needed on HM composition and infant growth, anthropometry, and nutritional status, a conclusion that echoes the collective voices of other HM scientists [64].

### Avenues for future research: moving beyond scientific reductionism

HM is a complex biological fluid containing hundreds of nutritive and non-nutritive components [2]; yet, nearly all studies included in this review measured associations between *individual* HM micronutrients and infant anthropometrics. This reductionist approach fails to address the complex composition of HM. For example, it is well established that Ca and phosphorus together mineralize bone and that bone matrix formation also requires protein [64,65]. Therefore, isolating Ca as a determinant of linear growth independent of protein and phosphorus might not be expected to lead to meaningful results except in the context of severe deficiency such as rickets [66]. Additionally, some micronutrients, though regulated independently, are secreted in predictable ratios in HM. For example, the median ratio of Ca and phosphorus is 1.7 in both preterm and term HM [65]. Both Ca and phosphorus concentrations in HM are highest during early postpartum and decrease gradually during lactation [[66], [67], [68], [69]]. Thus, high-quality research that employs systems biology approaches [70] is required to understand how HM micronutrients and other components work independently and together to influence infant growth and development.

Moreover, infants’ diets change dramatically in the first 2 y of life, from (ideally) exclusively HM in the first 6 mo to a progressively complex diet with declining proportions of nutrients coming from HM. For example, Zn and Fe concentrations in HM are highest during early lactation and gradually decline [[71], [72], [73]] until, by around 6 mo, HM alone is unable to meet the estimated daily requirements for these nutrients [74,75]. Multiple micronutrient concentrations follow similar patterns over lactation, making it difficult to disentangle, which is more important for growth outcomes, particularly when they are studied in isolation.

Weaning also means that complementary foods provide incrementally more nutrients than HM. Little is known about the bioavailability or absorption of nutrients from HM compared with complementary foods and whether the food matrix and/or infant physiology act as effect modifiers. These complexities underscore the need for large multidisciplinary studies that adequately measure dietary intake over the course of lactation, collect relevant biological samples from mother-infant dyads, and capture robust growth and development outcomes.

### Strengths and limitations

Strengths of our systematic review included the use of a registered protocol and a comprehensive, peer-reviewed search strategy. We comprehensively synthesized available evidence for all micronutrients in HM and all child anthropometrics in the first 2 y. The main limitation of our review was the inability to conduct meta-analyses due to the paucity of eligible studies and differences in sampling times, outcome measurements, and reporting practices. The individual studies included in our review also had limitations. Many did not provide results for all associations studied, reporting only the significant associations observed. Most studies only achieved a moderate quality score and were observational. Observational studies are subject to confounding bias, and most of the included studies did not adequately account for major confounders, including maternal BMI, birth anthropometrics, time postpartum, or infant diet (e.g., formula supplementation or complementary foods). Additionally, most studies measured HM micronutrient concentrations without measuring the volume of HM consumed, precluding them from estimating the *intake* of HM micronutrients. Moreover, nearly all studies failed to account for the complex composition of HM and only reported associations of individual nutrients.

## Conclusions

Even though HM is recommended as the exclusive food source for the first 6 mo of life and remains an important source of nutrition through 2 y, this systematic review reveals that the impact of HM micronutrients on the growth of healthy, term infants is woefully under-investigated. Current evidence suggests that HM iodine, Mn, Ca, and Zn may be positively correlated with infant growth, though these relationships remain largely unclear due to sparse data, small sample sizes, and methodological limitations of existing studies. Thus, the available evidence was largely inconclusive and failed to address the complex composition of HM. High-quality research that employs systems biology approaches [70] is required to understand how HM micronutrients and other components work independently and together to influence infant growth and development and to identify new avenues for future maternal, newborn, or infant nutritional interventions.

## Acknowledgments

We thank Nicole Askin, MLIS (WRHA Virtual Library, University of Manitoba), for peer review of the MEDLINE search strategy. We also thank the International Milk Composition Consortium (www.milcresearch.com/imic) members for fruitful discussions that helped inform the development of quality assessment and data extraction forms. Additionally, we thank the study authors who provided data for this review [25,35,37,44,46].

### Author contributions

The authors’ responsibilities were as follows – SMR, MBA: designed the research; SMR, MBA, MB, NR: oversaw the research; SMR, MB, JMM, DC, MG, RR, KKS, SM, CM, LL: conducted the systematic review; SMR, MB, LL, MBA: synthesized the data; SMR, MBA: wrote the paper and had primary responsibility for the final content; SMR, MB, JMM, MG, DTG, FJ, PK, LHA, DH, KGE: provided critical review and contribution to the manuscript; and all authors: read and approved the final manuscript.

### Conflict of interest

SMR, MB, and MBA have contributed to online courses on breast milk and the infant microbiome produced by Microbiome Courses. SMR is a former employee of Prolacta Bioscience and has also served as the scientific advisor for SimpliFed and a consultant for TraverseScience. Her contribution to this review occurred prior to these engagements. JMM has received support from the Bill & Melinda Gates Foundation and serves on the Council on Research for the American Academy of Nutrition and Dietetics. DC is supported by a Canadian Nurses Foundation Scholarship. DTG is funded by an unrestricted research grant from Medela AG. She is also currently funded by Telethon Child Health Grants and the Australian National Health and Medical Research Council. LHA has research grants from the Bill & Melinda Gates Foundation. MBA is supported by a Canada Research Chair and is a Canadian Institute for Advanced Research (CIFAR) Fellow in the Humans and the Microbiome Program; she has consulted for DSM and is a scientific advisor to TinyHealth. All other authors report no conflicts of interest.

### Funding

This review was undertaken as part of the International Milk Composition Consortium, funded by the Bill & Melinda Gates Foundation (INV-001734).

### Data availability

Data described in the manuscript, code book, and analytic code will be made available upon request pending application and approval by study authors.
