# Supplementary material for: Human Milk Micronutrients and Child Growth and Body Composition in the First 2 years: A Systematic Review
Source: Adv Nutr. 2023 Jun 13;15(1):100082. doi: 10.1016/j.advnut.2023.06.005 (PMC10831887; doi:10.1016/j.advnut.2023.06.005)
Supplement: Multimedia component 1 [file mmc1.pdf]

Table S1. Study quauality assessment criteria: human milk micronutrients and child growth.
Total score <7=Low, 7-13=Moderate, >13-17=High.

Item	Description	Points
**Human Milk Exposure Assessment**		<3=Low, 3-6=Moderate, >6-8=High
1	**Sampling strategy/ handling protocol**	
	**Fat-soluble vitamins (A, D, E, K)**	
1.1.0	Milk collection protocol	2 - Complete 24-hr collection*
		1 - Full breast expression standardized for time of day
		0 - Any other collection method
1.1.1	Stage of lactation	0.5 - Time postpartum standardized across participants
		0 - Time postpartum not standardized
1.1.2	Handling protocol	0.5 - Precautions taken to minimize light exposure
		0 - No precautions taken
	**Water-soluble vitamins (other than Biotin, Vitamins B-6 and B-12)**	
1.2.0	Milk collection protocol	1 - Full breast expression; morning and evening samples combined
		0.5 - Full breast expression only once, morning or night
		0 - Any other collection method
1.2.1	Timing of sample collection	1 - Avoids fasting and standardized standardized in relation to supplement use**
		0.5 - Avoids fasting or collection soon after vitamin supplement, not both
		0 - Fasting and supplemental use not taken into account
1.2.2	Stage of lactation	0.5 - Time postpartum standardized across participants
		0 - Time postpartum not standardized
	**Trace Minerals & Biotin**	
1.3.0	Milk collection protocol	1 - Full or partial expression (foremilk, midfeed, or hindmilk samples), standardized across women
		0 - Collection strategy not standardized across women
1.3.1	Timing of sample collection	1 - Time of day milk sampled standardized across women
		0 - Time of day milk sampled not standardized
1.3.2	Stage of lactation	0.5 - Time postpartum standardized across participants
		0 - Time postpartum not standardized
	**Vitains B-6 & B-12**	
1.4.0	Milk collection protocol	1 - Full or partial expression (foremilk, midfeed, or hindmilk samples), standardized across women
		0 - Collection strategy not standardized across women
1.4.1	Timing of sample collection	1 - Avoids fasting and standardized standardized in relation to supplement use**
		0.5 - Avoids fasting or collection soon after vitamin supplement, not both
		0 - Fasting and supplemental use not taken into account
1.4.2	Stage of lactation	0.5 - Time postpartum standardized across participants
		0 - Time postpartum not standardized
2	**Sample preparation**	
2.1.0	Reproducibility	1 - Reproducible using information provided in current paper OR direct reference
		0 - Not reproducible using informaiton provided in current paper OR direct reference
3	**Analytical Method**	
3.1.0	Validation / quality control	2 - Method validated in human milk, including recovery data and inter-/intra-assay variation*** in current paper OR direct reference
		1 - Particial validation (e.g., precision or accuracy data reported, but not both in current paper OR direct reference
		0.5 - Commercial assays or commonly used methods but no validation data specific to human milk in current paper OR direct reference
4	**Longitudinal sampling**	
4.1.0	Longitudinal sampling of human milk	1 - Milk samples collected longitudinally (e.g., 1, 3, and 6 mo)
		0 - Milk samples NOT collected longitudinally
5	**Human milk volume intake**	
5.1.0	Infant intake of human milk	1 - Volume of human milk infants consumed measured or estimated
		0 - Volume not considered
**Confounders Considered**		<3=Low, 3-4=Moderate, >4-5=High
6	**Infant diet**	
6.1.0	Breastfeeding exclusivity	2 - All infants exclusively breastfed at time of human milk collection
		up to 1.5 - If NOT all infants exclusively breastfed:
		0.5 - Reporting breastfeeding status
		0.5 - Adjusting for it in analyses
		0.5 - Reports number of proportion of human milk vs. other milks
		0 - Breastfeeding exclusivity of infants unclear

7	**Birth anthropometrics**	
7.1.0	Birth anthropometrics	1 - Any birth anthropometrics accounted for in study design or analyses
		0 - No birth anthropometrics accounted for
8	**Baseline characteristics**	
8.1.0	Maternal characteristics reported	0.5 - Reports maternal age parity, BMI, ethnicity, time postpartum, others relevant to study
		0 - Does not report maternal characteristics
8.1.1	Infant characteristics reported	0.5 - Reports infants age [or time postpartum] and sex
		0 - Does not report infant characteristics
8.1.2	Maternal characteristics accounted for	1 - Accounted for in analyses or study design
		0 - Not accounted for
8.1.3	Infant characteristics accounted for in analyses	1 - Accounted for in analyses or study design
		0 - Not accounted for
**Infant Anthropometric Outcomes**		<2=Low, 2-3=Moderate, >3-4=High
9	**Infant anthropometrics**	
9.1.0	Source of anthropometric measurements	1 - Trained staff
		0.5 - Collected from clinical records
		0 - Self-reported
9.1.1	Technical replicates performed?	1 - Yes
		0 - No
9.1.2	Longitudinal measurements?	1 - Infant anthropometrics measured multiple times over time (e.g., 1, 3, and 6 mo)
		0 - Anthropometrics NOT measured longitudinally
9.1.3	Timing of measurements across infants	1 - All infants measured at same age (± 1 week)
		2 - All infants measured at similar age (± 1 mo)
		0 - Timing of measurements not standardized across infants

*weighted combined aliquots of fore- and hind milk12 from one breast at each pumping over 24- hours (2 point).
(e.g., not collected soon after supplement use). *(e.g., data accuracy and precision)

Table S2. Study quality* assessment results: human milk micronutrients and child growth.

Study Details	Human Milk Exposure Assessment					Confounders Considered				Infant Anthropotmetric Outcomes				Summary & Total Scores**			
	1	2	3	4	5	6	7	8		9							
**Authors** (Alphabetical) **Country, Publication Year (Setting)**	**Sampling strategy/ handling protocol**	**Sample preparation**	**Analytical method**	**Longitudinal sampling**	**Human milk volume intake**	**Infant diet**	**Birth anthropometrics**	**Baseline characteristics reported**	**Baseline characteristics accounted for**	**Source of measurements**	**Techinical replicates**	**Longitudinal measurements**	**Standardization of timing of measurements**	**Human Milk Exposure Assessment**	**Confounders Considered**	**Infant Anthropometric Outcomes**	**TOTAL**
Bilston-John et al. Australia, 2021	3	1	2	1	1	1.5	0.5	1	0.25	0.5	0	1	0.75	8.0	3.3	2.3	**13.5**
Butte et al. USA, 1987	1.5	1	2	0.5	1	0	0	0.5	0	0.5	0	1	1	6.0	0.5	2.5	**9.0**
Casterline et al. Guatemala, 1997	2.25	1	2	0	0	2	0	0.5	0	1	0.5	0.5	0.5	5.3	2.5	2.5	**10.3**
Cisse et al. Senegal, 2002	2	1	0.5	0	1	0	0	0.375	0	0.5	0	0	1	4.8	0.4	2.3	**6.4**
Dorea Brazil, 1993	3	1	0.25	1	0	1.25	0.5	0.375	0.5	0.5	0	1	1	5.3	2.6	2.5	**10.4**
Doneray et al. Turkey, 2017	2	1	2	1	0	0	0.5	0.625	0.5	1	0	1	1	6.0	1.6	3.0	**10.6**
Dumrongwongsiri et al. Thailand, 2022	2	0.5	0.25	1	1	1.5	1	1	1	0.5	0	1	1	4.8	4.5	2.5	**11.8**
Ellsworth et al. USA, 2020	3	1	2	1	0	2	1	1	1	0.75	0	1	0.75	7.0	5.0	2.5	**14.5**
Han et al. Korea, 2009	2	0.5	1.25	1	0.5	0.75	0.5	0.75	1	1	0	1	0.75	5.3	3.0	2.8	**10.5**
Han et al. Korea, 2011	1	0.5	1.5	1	0.75	0.75	0.5	0.5	0	1	0	1	0.75	4.8	1.8	2.8	**9.3**
Han et al. Korea, 2022	1	1	0.5	0	0	1.5	0.5	0.75	1	1	0.5	0	1	2.5	3.8	2.5	**8.8**
Hussein et al. Egypt, 1987	0.5	1	0.5	0	0	0.5	0	0	0.5	1	0	0.5	0	2.0	1.0	1.5	**4.5**
Jarjou et al. The Gambia, 2012	3	1	2	1	1	1.5	0.75	0.5	0.5	1	0	1	0.75	8.0	3.3	2.8	**14.0**
Kang-Yoon et al. USA, 1992	2	1	2	0.5	1	0.5	1	0.375	0.5	1	0	1	0.5	6.5	2.4	2.5	**11.4**
Krebs et al. USA, 1994	2	1	2	1	1	0.5	1	1	0.5	1	1	1	0.5	7.0	3.0	3.5	**13.5**
Li et al. Guatemala, 2016	2	1	1	0.5	0.5	0.75	0	0.75	1	1	0.5	0.5	1	5.0	2.5	2.0	**9.5**
Li et al. Guatemala, 2019	1.5	1	1	1	0	0.75	0	0.5	1	1	0.5	1	0.5	4.5	2.3	3.0	**9.8**
Mahdavi et al. Iran, 2010	1.5	1	1.5	0	0	2	1	0.75	0.5	1	0	0	0.25	4.0	4.3	1.3	**9.5**
Minato et al. Japan, 2019	1	1	0.5	1	0	0.75	1	0.375	0.5	0.5	0	1	1	3.5	2.6	2.5	**8.6**
Motoyama et al. Japan, 2021	1	1	0.75	1	0	0.5	0.5	0.75	0	0.5	1	1	0.25	3.8	1.8	2.8	**8.3**
Nazeri et al. Iran, 2020	2	0.75	2	0	0	1	1	0.875	1	1	0	0.5	0.75	4.8	3.9	2.3	**10.9**
Nikniaz et al. Iran, 2019	2	1	1.5	1	0	0.5	0	1	0.5	1	0	1	0.5	5.5	2.0	2.5	**10.0**
Palmer et al. Zambia, 2016	0.75	1	2	1	0	0.25	0	0.5	0.5	1	0	1	0.25	4.8	1.3	2.3	**8.3**
Salmenpera et al. Finland, 1994	2.5	1	1.5	1	1	0.5	0	0	0	1	0	1	0.25	7.0	0.5	2.3	**9.8**
Samuel et al. India, 2014	3	1	2	1	1	1.5	1	1	1	1	1	1	0.75	8.0	4.5	3.8	**16.3**
Sievers et al. Germany, 1992	1	1	2	1	1	2	0.5	0.75	0.25	1	0.5	1	1	6.0	3.5	3.5	**13.0**
Umeta et al. Ethiopia, 2003	2	1	1	0	0	0.5	0	1	0.25	1	0.5	0	0.5	4.0	1.8	2.0	**7.8**
Young et al. Guatemala, India, Pakistan, 2021	1	0.5	0.75	0	0	0	0.5	0.5	0.25	1	0	1	0.75	2.3	1.3	2.8	**6.3**

*See Table S1 for 9 categories of quality assessment criteria. **Red = low, Yellow = moderate, Green = high. See Methods for details.
World Bank Income Setting: LMIC, low middle income country; UMIC, upper middle income country; HIC, high income country.

Table S3. Characteristics and results of included studies reporting on human milk micronutrients and infant anthropometrics - organized by nutrient.

Authors, country, publication year (income setting)	Design and participants	Timing of milk sampling	Timing of infant anthropometrics	Estimated intake or HM concentration*	Anthropometric outcome measures and standards	Associations**	Major confounders considered
**Vitamin A (retinol)**							
Hussein et al.<br>Egypt, 1987<br>(LMIC)	Cross-sectional<br>14 healthy (35 total, including ill infants)	5 to 53 wks postpartum	5 to 53 wks postpartum	Concentration in HM<br>29.5 ± 4.92 ug/100 g (healthy dyads)	arc, sin of the square root of (weight/height^2-A)	(+) Associations between retinal and body mass index	None
Palmer et al.<br>Zambia, 2016*<br>(LMIC)	Longitudinal<br>149; 49 (white maize + placebo)<br>50 (orange maize + placebo)<br>50 (white maize + vitamin A)	4-12 mo (once pre- and post-intervention)	4-12 mo (once pre- and post-intervention)	Concentration in HM<br>1.1 ± 0.7 μmol/L	weight, length	(No) Associations between vitamin A and pro-vitamin A and infant weight, length	None
**Pro-vitamin A (beta-carotene)**							
Palmer et al.<br>Zambia, 2016*<br>(LMIC)	Longitudinal<br>149; 49 (white maize + placebo)<br>50 (orange maize + placebo)<br>50 (white maize + vitamin A)	4-12 mo (once pre- and post-intervention)	4-12 mo (once pre- and post-intervention)	Concentration in HM<br>10.9 ± 8.0 mmol/L	weight, length	(No) Associations between beta-carotene A and pro-vitamin A and infant weight, length	None
**Vitamin B2 (riboflavin)**							
Young et al.<br>Guatemala, India, Pakistan, 2021<br>(LMIC)	RCT<br>200	2 wks	1, 3, and 6 mo postpartum	Concentration	LAZ-slope, WAZ-slope, WLZ-slope	(No) Association between Riboflavin (B2) and LAZ, WAZ or WLZ	Maternal pre-conception BMI category, site, and arm
**Vitamin B3 (niacin)**							
Young et al.<br>Guatemala, India, Pakistan, 2021<br>(LMIC)	RCT<br>200	2 wks	1, 3, and 6 mo postpartum	Concentration	LAZ-slope, WAZ-slope, WLZ-slope	(No) Association between Niacin (B3) and LAZ, WAZ or WL	Maternal pre-conception BMI category, site, and arm
**Vitamin B6**							
Kang-Yoon et al.<br>USA, 1992<br>(HIC)	Supplemental Trial<br>20;<br>7 (mothers supplemented with 2 mg PN-HCl);<br>7 (mothers supplemented w/ 2 mg PN-HCl + infants supplemented with 0.4 mg PN-HCl);<br>6 (mothers supplemented with 27 mg PN-HCl)	7 days (foremilk)	7, 14, 28 days	Estimated intake from HM<br>Data not extractable	weight gain	(+) Associations between vitamin B6 and weight gain before adjustment for covariates	Birth weight, plasma pyridoxal-5'-phosphate
Young et al.<br>Guatemala, India, Pakistan, 2021<br>(LMIC)	RCT<br>200	2 wks	1, 3, and 6 mo postpartum	Concentration	LAZ-slope, WAZ-slope, WLZ-slope	(No) Association between B6 and LAZ, WAZ or WLZ	Maternal pre-conception BMI category, site, and arm
**Vitamin B12**							
Casterline et al.<br>Guatemala, 1997<br>(LMIC)	Cross-sectional<br>113	3 mo	3 mo	Concentration in HM<br>689.7 ± 490.7 pmol/L	WAZ, HAZ, WHZ	(No) Associations between vitamin B12 and WAZ, HAZ, WHZ	None
Young et al.<br>Guatemala, India, Pakistan, 2021<br>(LMIC)	RCT<br>200	2 wks	1, 3, and 6 mo postpartum	Concentration	LAZ-slope, WAZ-slope, WLZ-slope	(No) Association between B12 and LAZ, WAZ or WLZ	Maternal pre-conception BMI category, site, and arm
**Biotin**							
Young et al.<br>Guatemala, India, Pakistan, 2021<br>(LMIC)	RCT<br>200	2 wks	1, 3, and 6 mo postpartum	Concentration	LAZ-slope, WAZ-slope, WLZ-slope	(No) Association between Biotin and LAZ, WAZ or WLZ	Maternal pre-conception BMI category, site, and arm
**Choline**							
Young et al.<br>Guatemala, India, Pakistan, 2021<br>(LMIC)	RCT<br>200	2 wks	1, 3, and 6 mo postpartum	Concentration	LAZ-slope, WAZ-slope, WLZ-slope	(No) Association between Choline and LAZ, WAZ or WLZ	Maternal pre-conception BMI category, site, and arm
**Folate**							
Han et al.<br>Korea, 2009*<br>(LMIC)	Longitudinal<br>20;<br>12 females<br>8 males	1, 2, 4, 5 and 6 mo	1, 2, 4, 5, 6 and 12 mo	Concentration in HM<br>Data not extractable	weight, length	(No) Associations between folate and weight or length	None
**Pantothenic Acid**							
Young et al.<br>Guatemala, India, Pakistan, 2021<br>(LMIC)	RCT<br>200	2 wks	1, 3, and 6 mo postpartum	Concentration	LAZ-slope, WAZ-slope, WLZ-slope	(No) Association between Pantothenic acid and LAZ, WAZ or WLZ	Maternal pre-conception BMI category, site, and arm
**Thiamin**							
Young et al.<br>Guatemala, India, Pakistan, 2021<br>(LMIC)	RCT<br>200	2 wks	1, 3, and 6 mo postpartum	Concentration	LAZ-slope, WAZ-slope, WLZ-slope	(No) Association between Thiamin and LAz, WAZ or WLZ	Maternal pre-conception BMI category, site, and arm
**Calcium (Ca)**							
Bilston-John et al,<br>Australia, 2021 (HIC)	Cohort<br>83	1, 2, 3, 4, 5, 6, 9, and 12 mo postpartum	3 and 6 mo postpartum	Concentration and estimated intake	weight, length, HC, LFAZ, HCFAZ, WFAZ<br>(WHO Standards)	(No) Association between any micro or macro minterals and infants anthropometrics	None

Table S3. Characteristics and results of included studies reporting on human milk micronutrients and infant anthropometrics - organized by nutrient.

Authors, country, publication year (income setting)	Design and participants	Timing of milk sampling	Timing of infant anthropometrics	Estimated intake or HM concentration*	Anthropometric outcome measures and standards	Associations**	Major confounders considered
Butte et al.<br>USA, 1987	Longitudinal<br>45	1,2,3,4 mo	1, 2, 3, 4 mo	Estimated intake from HM<br>294 ± 35 μg/g	weight, weight-for-age percentile, growth rate (g/d)	(+) Associations between Ca and weight, weight-for-age, growth	None
Cisse et al.<br>Senegal, 2002*	Longitudinal<br>133;<br>41 Mothers supplemented with millet (high protein)<br>35 Mothers supplemented with maize (high CHO)<br>57 Unsupplemented mothers	14 days	3 mo	Concentration in HM<br>235 ± 40 to 260 ± 37 mg/L	weight-for-length; length-for-age (percentiles)	(+) Association between Ca and weight-for-length, length-for-age (all groups)	None
Jarjou et al.<br>The Gambia, 2012	Longitudinal<br>30;<br>17 females<br>13 males	3 and 12 mo	3 and 12 mo	Concentration in HM<br>203 ± 27 mg/L (3 mo)<br>152 ± 24 (12 mo)	weight, length, bone mineral content (BMC), bone width (BW), and bone mineral density (BMD)	(+) Associations between Ca and weight, length<br>(No) Associations between Ca and bone mineral content, width, or density	Infant sex; BMC model adjusted for bone width, weight and length to give a size-adjusted BMC
Li et al.<br>Guatemala, 2019<br>(LMIC)	Longitudinal<br>114;<br>80 Mothers without subclinical mastitis (non-SCM) during early lactation (2-46 days)<br>34 with SCM in early lactation (2-46 days)<br>103 non-SCM in established lactation (4-6 mo)<br>11 with SCM in established lactation (4-6 mo)	Early (2-46 days) and established lactation (4-6 mo)	Early (2-46 days) and established lactation (4-6 mo)	Concentration in HM<br>6.69 ± 1.15 mmol/L (early, non-SCM)<br>6.54 ± 1.49 (early, SCM)<br>6.18 ± 0.78 (established, Non-SCM)<br>6.22 ± 1.35 (established, SCM)	WAZ, LAZ, WLZ, HCAZ,at early lactation and with growth velocity (change in infant growth/d) for weight, length, and HC between early and established lactation	(No) Assumed associations between Ca and WAZ, LAZ, WLZ, HCAZ, or growth velocity (weight, length, HC)	**Early lactation models:** somatic cell count, milk cytokine concentrations, estimated milk intake<br><br>**Growth trajectory models:** variables from early lactation models, plus infant sex, early length or HC, maternal weight, height, and mixed feeding (yes/no)
Minato et al.<br>Japan, 2019<br>(HIC)	Longitudinal<br>56 (1 mo)<br>42 (3 mo)	1 and 3 mo	1 and 3 mo	Concentration in HM<br>Median (IQR): 29.8 (26.7, 33.3) mg/L (1 mo)<br>28.7 (25.9, 31.9) (3 mo)	weight, length	(No) Associations between Ca and infant weight or length at 1 or 3 mo	None
Umeta et al.<br>Ethiopia, 2003<br>(LMIC)	Cross-sectional<br>305 (253 analyzed; n=92 stunted; 161 nonstunted)	1 collection time between 5-11 mo	1 sampling time between 5-11 mo	Concentration in HM<br>Mothers of nonstunted infants, mean (SEM): 0.35 (0.01) mmol/L	stunting<br>(NCHS)	(No) Associations among milk calcium and stunting	Infant age
**Phosphorus (P)**							
Butte et al.<br>USA, 1987<br>(HIC)	Longitudinal<br>45	1,2,3,4 mo	1, 2, 3, 4 mo	Estimated intake from HM<br>139 ± 19 μg/g	weight, weight-for-age percentile, growth rate (g/d)	(+) Associations between P and weight, weight-for-age, growth rate<br>(No) Assumed associations for all other combinations of P and infant anthropometrics	None
Minato et al.<br>Japan, 2019<br>(HIC)	Longitudinal<br>56 (1 mo)<br>42 (3 mo)	1 and 3 mo	1 and 3 mo	Concentration in HM<br>Median (IQR): 16.6 (14.6, 18.3) mg/L (1 mo)<br>13.4 (11.6, 14.4) (3 mo)	weight, length	(No) Associations between P and infant weight, length at 1 or 3 mo	None
**Potassium (K)**							
Bilston-John et al,<br>Australia, 2021<br>(HIC)	Cohort<br>83	1, 2, 3, 4, 5, 6, 9, and 12 mo postpartum	3 and 6 mo postpartum	Concentration and estimated intake	weight, length, HC, LFAZ, HCFAZ, WFAZ<br>(WHO Standards)	(No) Association between any micro or macro minerals and infants anthropometrics	None
Butte et al.<br>USA, 1987<br>(HIC)	Longitudinal<br>45	1,2,3,4 mo	1, 2, 3, 4 mo	Estimated intake from HM<br>443 ± 59 μg/g	weight, weight-for-age percentile, growth rate (g/d)	(+) Associations between K and weight, weight-for-age<br>(No) Assumed associations for all other combinations of K and weight at 3-4 mo, weight-for-age at 1-2 mo, growth rate	None
Cisse et al.<br>Senegal, 2002*<br>(LMIC)	Longitudinal<br>133;<br>41 Mothers supplemented with millet<br>35 Mothers supplemented with maize<br>57 Unsupplemented mothers	14 days	3 mo	Concentration in HM<br>501 ± 78 mg/L (Millet)<br>481 ± 78 (Corn)<br>509 ± 74 (Unsupplemented)	weight-for-length; length-for-age (percentiles)	(+) Association between K and weight-for-length, length-for-age (all groups)	None
Li et al.<br>Guatemala, 2019<br>(LMIC)	Longitudinal<br>114;<br>80 Mothers without subclinical mastitis (non-SCM) during early lactation (2-46 days)<br>34 with SCM in early lactation (2-46 days)<br>103 non-SCM in established lactation (4-6 mo)<br>11 with SCM in established lactation (4-6 mo)	Early (2-46 days) and established lactation (4-6 mo)	Early (2-46 days) and established lactation (4-6 mo)	Concentration in HM<br>Data unavailable	WAZ, LAZ, WLZ, HCAZ,at early lactation and with growth velocity (change in infant growth/d) for weight, length, and HC between early and established lactation	(No) Assumed associations between K and WAZ, LAZ, WLZ, HCAZ, or growth velocity (weight, length, HC)	**Early lactation models:** somatic cell count, milk cytokine concentrations, estimated milk intake<br><br>**Growth trajectory models:** variables from early lactation models, plus infant sex, early length or HC, maternal weight, height, and mixed feeding (yes/no)
**Magnesium (Mg)**							
Bilston-John et al,<br>Australia, 2021<br>(HIC)	Cohort<br>83	1, 2, 3, 4, 5, 6, 9, and 12 mo postpartum	3 and 6 mo postpartum	Concentration and estimated intake	weight, length, HC, LFAZ, HCFAZ, WFAZ<br>(WHO Standards)	(No) Association between any micro or macro minerals and infants anthropometrics	None
Butte et al.<br>USA, 1987<br>(HIC)	Longitudinal<br>45	1,2,3,4 mo	1, 2, 3, 4 mo	Estimated intake from HM<br>30 ± 6 μg/g	weight, weight-for-age percentile, growth rate (g/d)	(+) Associations between Mg and weight, weight-for-age<br>(No) Assumed associations for Mg and other infant anthropometrics	None
Cisse et al.<br>Senegal, 2002*<br>(LMIC)	Longitudinal<br>133;<br>41 Mothers supplemented with millet<br>35 Mothers supplemented with maize<br>57 Unsupplemented mothers	14 days	3 mo	Concentration in HM<br>31 ± 7 mg/L (Millet)<br>34 ± 5 (Corn)<br>32 ± 7 (Unsupplemented)	weight-for-length (percentile)	(+) Association Mg and weight-for-length (all groups)	None
Li et al.<br>Guatemala, 2019<br>(LMIC)	Longitudinal<br>114;<br>80 Mothers without subclinical mastitis (non-SCM) during early lactation (2-46 days)<br>34 with SCM in early lactation (2-46 days)<br>103 non-SCM in established lactation (4-6 mo)<br>11 with SCM in established lactation (4-6 mo)	Early (2-46 days) and established lactation (4-6 mo)	Early (2-46 days) and established lactation (4-6 mo)	Concentration in HM<br>0.92 ± 0.18 mmol/L (early, non-SCM)<br>1.03 ± 0.23 (early, SCM)<br>1.43 ± 0.21 (established, non-SCM)<br>1.45 ± 0.22 (established, SCM)	WAZ, LAZ, WLZ, HCAZ,at early lactation and with growth velocity (change in infant growth/d) for weight, length, and HC between early and established lactation	(-) Associations bewteen Mg and linear growth velocity<br>(No) Assumed associations between Mg and WAZ, LAZ, WLZ, HCAZ or change in weight or HC	**Early lactation models:** somatic cell count, milk cytokine concentrations, estimated milk intake<br><br>**Growth trajectory models:** variables from early lactation models, plus infant sex, early length or HC, maternal weight, height, and mixed feeding (yes/no)

Table S3. Characteristics and results of included studies reporting on human milk micronutrients and infant anthropometrics - organized by nutrient.

Authors, country, publication year (income setting)	Design and participants	Timing of milk sampling	Timing of infant anthropometrics	Estimated intake or HM concentration*	Anthropometric outcome measures and standards	Associations**	Major confounders considered
**Sodium (Na)**							
Bilston-John et al, Australia, 2021 (HIC)	Cohort 83	1, 2, 3, 4, 5, 6, 9, and 12 mo postpartum	3 and 6 mo postpartum	Concentration and estimated intake	weight, length, HC, LFAZ, HCFAZ, WFAZ<br><br>(WHO Standards)	(No) Association between any micro or macro minterals and infants anthropometrics	None
Butte et al. USA, 1987 (HIC)	Longitudinal 45	1,2,3,4 mo	1, 2, 3, 4 mo	Estimated intake from HM 112 ± 33 µg/g	weight, weight-for-age percentile, growth rate (g/d)	(No) Assumed associations for Na and infant anthropometrics	None
Cisse et al. Senegal, 2002* (LMIC)	Longitudinal 133;<br>41 Mothers supplemented with millet (high protein)<br>35 Mothers supplemented with maize (high CHO)<br>57 Unsupplemented mothers	14 days	3 mo	Concentration in HM<br>136 ± 59 mg/L (Millet)<br>138 ± 43 (Corn)<br>132 ± 67 (Unsupplemented)	weight-for-length (percentile)	(+) Association between Na and weight-for-length (millet and maize supplemented groups only)	None
**Zinc (Zn)**							
Bilston-John et al, Australia, 2021 (HIC)	Cohort 83	1, 2, 3, 4, 5, 6, 9, and 12 mo postpartum	3 and 6 mo postpartum	Concentration and estimated intake	weight, length, HC, LFAZ, HCFAZ, WFAZ<br><br>(WHO Standards)	(No) Association between any micro or macro minterals and infants anthropometrics	None
Butte et al. USA, 1987 (HIC)	Longitudinal 45	1,2,3,4 mo	1, 2, 3, 4 mo	Estimated intake from HM 1.5 ± 0.8 µg/g	weight, weight-for-age percentile, growth rate (g/d)	(No) Assumed associations for Zn and infant anthropometrics	None
Cisse et al. Senegal, 2002* (LMIC)	Longitudinal 133;<br>41 Mothers supplemented with millet (high protein)<br>35 Mothers supplemented with maize (high CHO)<br>57 Unsupplemented mothers	14 days	3 mo	Concentration in HM<br>Unsupplemented<br>Mean (SD): 1.3 ± 0.5 mg/L	weight-for-length (percentile)	(+) Association between Zn and weight-for-length (millet and maize supplemented groups only)	None
Doneray et al. Turkey, 2017 (LMIC)	Longitudinal 37;<br>25 females<br>12 males	PP 8-12 days, PP 25-30 days, 2 collections each (foremilk and hindmilk)	PP 8-12 days, pp 25-30 days	Concentration in HM<br>455.2 ± 215.1 µg/dL (early, hindmilk)<br>336.1 ± 235.1 µg/dL (established, hindmilk)	weight	(-) Associations between Zn (hindmilk) and weight during early lactation<br>(+) Associations between Zn (hindmilk) and weight during established lactation	None
Dorea Brazil, 1993 (LMIC)	Cohort 8;<br>3 females;<br>5 males	0, 1, 2, 3, 4, 5, 6 mo	0, 1, 2, 3, 4, 5, 6 mo	Concentration in HM<br>Data not extractable	Ponderal growth, linear growth (WHO Child Growth Standards)	(+) Associations between Zn and Ponderal growth and linear growth in the first 6 mo	Adjusted for total nitrogen and fat
Dumrongwongsiri et al. Thailand, 2022 (LMIC)	Cohort<br><br>120 enrolled (64 and 56 analysed)	2 and 4 mo postpartum	2 and 4 mo postpartum	Concentration and estimated intake	Infant weight, height, weight gain, lenghth gain, WFA-Z, WAZ, LFAZ, LAZ, WFLZ, WLZ<br><br>(WHO Standards)	(+) Association between HM Zn and weight gain (birth - 4 mo) and WLZ<br>(No) Association between HM Zn and length gain or LAZ	Age, anthropometric measurement and dietary intakes
Han et al. South Korea, 2011* (LMIC)	Longitudinal 20;<br>12 females<br>8 males	1, 2, 4, 5 and 6 mo	1, 2, 4, 5, and 6 (note, anthros measured until 36 mo, but correlations with human milk available only through 6 mo)	Estimated intake from HM<br>37.5 µmol/L (1 mo)<br>23.5 (2 mo)<br>15.4 (4 mo)<br>14.2 (5 mo)<br>14.0 (6 mo)	weight, length	(-) Association between Zn intake from HM and weight at 1 mo only<br>(No) Association between Zn intake from HM and length	None
Krebs et al. USA, 1994 (HIC)	Longitudinal 71;<br>40 females<br>31 males	2 wks, 1,2,3,4,5,6,7 mo; females followed through 9 mo	2 wks, 1,2,3,4,5,6,7 mo; females followed through 9 mo	Estimated intake from HM<br>2.30 ± 0.68 g/d (2 wk)<br>1.00 ± 0.43 (3 mo)<br>0.81 ± 0.42 (5 mo)<br>0.52 ± 0.31 (7 mo)	WAZ, WLZ, HCAZ	(+) Associations between Zn and WAZ, WLZ, HCAZ, but only at distinct times	Milk volume intake
Li et al. Guatemala, 2019 (LMIC)	Longitudinal 114;<br>80 Mothers without subclinical mastitis (non-SCM) during early lactation (2-46 days)<br>34 with SCM in early lactation (2-46 days)<br>103 non-SCM in established lactation (4-6 mo)<br>11 with SCM in established lactation (4-6 mo)	Early (2-46 days) and established lactation (4-6 mo)	Early (2-46 days) and established lactation (4-6 mo)	Concentration in HM<br>66.4 ± 64.9 µmol/L (early, non-SCM)<br>60.3 ± 47.8 (early, SCM)<br>19.1 ± 9.8 (established, non-SCM)<br>18.2 ± 9.1 (established, SCM)	WAZ, LAZ, WLZ, HCAZ,at early lactation and with growth velocity (change in infant growth/d) for weight, length, and HC between early and established lactation	(-) Associations between Zn and HCAZ<br>(No) Assumed associations between Zn and WAZ, LAZ, WLZ, or growth velocity	**Early lactation models:** somatic cell count, milk cytokine concentrations, estimated milk intake<br><br>**Growth trajectory models:** variables from early lactation models, plus infant sex, early length or HC, maternal weight, height, and mixed feeding (yes/no)
Mahdavi et al. Iran, 2010 (LMIC)	Cross-sectional 182;<br>91 Urban<br>35 females;<br>56 males<br>91 Rural<br>35 females;<br>56 males	90-120 days	90-120 days	Concentration in HM<br>1.85 ±0.5 mg/L	WAZ, HAZ	(+) Associations between Zn and WAZ, but only among infants whose mothers with high values of milk Zn<br>(No) Associations between Zn and HAZ	Rural vs. urban setting; maternal BMI, height, dietary energy intake; infant birth weight
Motoyama et al. Japan, 2021 (HIC)	Cohort<br><br>129 enroleed (79 analysed)	1 mo and 3 mo	1 mo and 3 mo	Concentration in HM	weight, height, HC (no standards)	(No) Association between Zn and infant length, weight or head circumferencN	none

Table S3. Characteristics and results of included studies reporting on human milk micronutrients and infant anthropometrics - organized by nutrient.

Authors, country, publication year (income setting)	Design and participants	Timing of milk sampling	Timing of infant anthropometrics	Estimated intake or HM concentration*	Anthropometric outcome measures and standards	Associations**	Major confounders considered
Salmenpera et al.<br>Finland, 1994<br>(HIC)	Longitudinal<br>200	2, 4, 6, 7.5 mo	2, 4, 6, 7.5 mo	Both<br>4.75 (3.27, 6.90) mg/L (0 mo)<br>1.41 (1.10, 2.19) (2 mo)<br>0.90 (0.58, 1.38) (4 mo)<br>0.67 (0.40, 1.13) (6 mo)<br>0.61 (0.39, 0.97) (7.5 mo)	weight, length, weight- and length-squared index, subscapular and bicep skinfold thickness	(+) Associations between Zn and weight velocities<br><br>(No) Associations between Zn intake nor milk Zn concentration and weight, length, or length-squared index, skinfold thickness	None
Samuel et al.<br>India, 2014<br>(LMIC)	Longitudinal<br>58;<br>50 followed through 6 mo	1, 3, 6 mo	1, 3, 6 mo	Estimated intake from HM<br>2.54 (1.83, 3.30) (1 mo)<br>1.35 (0.95, 1.74) (3 mo)<br>1.06 (0.85, 1.63) (6 mo)	weight, length	(No) Associations between Zn and weight and length gain	Non-breast milk water intake, infant age, gender, and infant weight and length at birth and mo 3
Sievers et al.<br>Germany, 1992<br>(HIC)	Longitudinal<br>10	45 collection times during first 17 wks	45 collection times during first 17 wks	Concentration in HM<br>3.56 (2.4, 4.9) mg/L (17 d)<br>2.6 (1.6, 3.6) (35 d)<br>7 (1.1, 2.8) (56 d)<br>1.34 (0.8, 1.38) (85 d)<br>1.2(0.64-1.9) (117 d)	weight	(-) Associations between Zn and weight	Non-breast milk water intake, infant age, gender, and infant weight and length at birth and mo 3
Umeta et al.<br>Ethiopia, 2003<br>(LMIC)	Cross-sectional<br>305 (253 analyzed; n=92 stunted; 161 nonstunted)	1 collection time between 5-11 mo	1 sampling time between 5-11 mo	Concentration in HM<br>10.4 ± 0.3 (SEM) µmol/L (unstunted)	stunting<br>(NCHS)	(+) Associations between low milk Zn and stunting	Infant age
**Iron (Fe)**							
Bilston-John et al,<br>Australia, 2021<br>(HIC)	Cohort<br>83	1, 2, 3, 4, 5, 6, 9, and 12 mo postpartum	3 and 6 mo postpartum	Concentration and estimated intake	weight, length, HC, LFAZ, HCFAZ, WFAZ<br><br>(WHO Standards)	(No) Association between any micro or macro minterals and infants anthropometrics	None
Butte et al.<br>USA, 1987<br>(HIC)	Longitudinal<br>45	1,2,3,4 mo	1, 2, 3, 4 mo	Estimated intake from HM<br>0.197 ±0.091 µg/g	weight, weight-for-age percentile, growth rate (g/d)	(No) Assumed associations for Fe and infant anthropometrics	None
Dumrongwongsiri et al.<br>Thailand, 2022<br>(LMIC)	Cohort<br><br>120 enrolled (64 and 56 analysed)	2 and 4 mo postpartum	2 and 4 mo postpartum	Concentration and estimated intake	Infant weight, height, weight gain, lenghth gain, WFA-Z, WAZ, LFAZ, LAZ, WFLZ, WLZ<br><br>(WHO Standards)	(No) Association between Fe and weight gain, length gain, WLZ or LAA	Age, anthropometric measurement and dietary intakes
Han et al.<br>Korea, 2011*<br>(LMIC)	Longitudinal<br>20;<br>12 females<br>8 males	1, 2, 4, 5 and 6 mo	1, 2, 4, 5, 6 and 12 mo	Estimated intake from HM<br>Data unavailable	weight, length	(No) Associations between Fe intake from HM and weight, length	None
Li et al.<br>Guatemala, 2019<br>(LMIC)	Longitudinal<br>114;<br>80 Mothers without subclinical mastitis (non-SCM) during early lactation (2-46 days)<br>34 with SCM in early lactation (2-46 days)<br>103 non-SCM in established lactation (4-6 mo)<br>11 with SCM in established lactation (4-6 mo)	Early (2-46 days) and established lactation (4-6 mo)	Early (2-46 days) and established lactation (4-6 mo)	Concentration in HM<br>6.95 ± 3.23 µmol/L (early, non-SCM)<br>8.97 ± 7.07 (early, SCM)<br>4.88 ± 3.84 (established, non-SCM)<br>6.76 ± 4.01 (established, SCM)	WAZ, LAZ, WLZ, HCAZ,at early lactation and with growth velocity (change in infant growth/d) for weight, length, and HC between early and established lactation	(No) Assumed associations between Fe and WAZ, LAZ, WLZ, HCAZ, or growth velocity	**Early lactation models:** somatic cell count, milk cytokine concentrations, estimated milk intake<br><br>**Growth trajectory models:** variables from early lactation models, plus infant sex, early length or HC, maternal weight, height, and mixed feeding (yes/no)
Mahdavi et al.<br>Iran, 2010<br>(LMIC)	Cross-sectional<br>182;<br>91 Urban<br>35 females;<br>56 males<br>91 Rural<br>35 females;<br>56 males	90-120 days	90-120 days	Concentration in HM<br>0.81 ± 0.2 mg/L (urban)<br>0.9 ±0.3 (rural)<br>0.85 ± 0.2 (total)	WAZ, HAZ	(No) Associations between Fe and WAZ or HAZ	Rural vs. urban setting; maternal BMI, height, dietary energy intake; infant birth weight
Motoyama et al.<br>Japan, 2021<br>(HIC)	Cohort<br><br>129 enroleed (79 analysed)	1 mo and 3 mo	1 mo and 3 mo	Concentration in HM	weight, height, HC<br>(no standards)	(No) Association between Fe and infant length, weight or head circumferencN	None
**Copper (Cu)**							
Bilston-John et al,<br>Australia, 2021<br>(HIC)	Cohort<br>83	1, 2, 3, 4, 5, 6, 9, and 12 mo postpartum	3 and 6 mo postpartum	Concentration and estimated intake	weight, length, HC, LFAZ, HCFAZ, WFAZ<br><br>(WHO Standards)	(No) Association between any micro or macro minterals and infants anthropometrics	None
Butte et al.<br>USA, 1987<br>(HIC)	Longitudinal<br>45	1,2,3,4 mo	1, 2, 3, 4 mo	Estimated intake from HM<br>0.308 ± 0.075 µg/g	weight, weight-for-age percentile, growth rate (g/d)	(No) Assumed associations for Cu and infanth anthropometrics	None
Han et al.<br>Korea, 2011*<br>(LMIC)	Longitudinal<br>20;<br>12 females<br>8 males	1, 2, 4, 5 and 6 mo	1, 2, 4, 5, 6 and 12 mo	Estimated intake from HM<br>Data unavailable	weight, length	(-) Associations between Cu intake from HM and weight at 1 and 5 mo<br>(No) Associations between Cu intake from HM and length	None

Table S3. Characteristics and results of included studies reporting on human milk micronutrients and infant anthropometrics - organized by nutrient.

Authors, country, publication year (income setting)	Design and participants	Timing of milk sampling	Timing of infant anthropometrics	Estimated intake or HM concentration*	Anthropometric outcome measures and standards	Associations**	Major confounders considered
Li et al.<br>Guatemala, 2019<br>(LMIC)	Longitudinal<br>114;<br>80 Mothers without subclinical mastitis (non-SCM) during early lactation (2-46 days)<br>34 with SCM in early lactation (2-46 days)<br>103 non-SCM in established lactation (4-6 mo)<br>11 with SCM in established lactation (4-6 mo)	Early (2-46 days) and established lactation (4-6 mo)	Early (2-46 days) and established lactation (4-6 mo)	Concentration in HM<br>7.76 ± 1.91 µmol/L (early, non-SCM)<br>8.22 ± 2.11 (early, SCM)<br>3.95 ± 1.17 (established, non-SCM)<br>4.89 ± 2.17 (established, SCM)	WAZ, LAZ, WLZ, HCAZ,at early lactation and with growth velocity (change in infant growth/d) for weight, length, and HC between early and established lactation	(No) Assumed associations between Cu and WAZ, LAZ, WLZ, HCAZ, or growth velocity	**Early lactation models:** somatic cell count, milk cytokine concentrations, estimated milk intake<br><br>**Growth trajectory models:** variables from early lactation models, plus infant sex, early length or HC, maternal weight, height, and mixed feeding (yes/no)
Mahdavi et al.<br>Iran, 2010<br>(LMIC)	Cross-sectional<br>182;<br>91 Urban<br>35 females;<br>56 males<br>91 Rural<br>35 females;<br>56 males	90-120 days	90-120 days	Concentration in HM<br>0.58 ± 0.4 mg/L (urban)<br>0.49 ± 0.2 (rural)<br>0.53 ± 0.3 (total)	WAZ, HAZ	(No) Associations between Cu, Fe and WAZ or HAZ	Rural vs. urban setting; maternal BMI, height, dietary energy intake; infant birth weight
Motoyama et al.<br>Japan, 2021<br>(HIC)	Cohort<br><br>129 enroleed (79 analysed)	1 mo and 3 mo	1 mo and 3 mo	Concentration in HM	weight, height, HC<br>(no standards)	(No) Association between Cu and infant length, weight or head circumferencN	None
Umeta et al.<br>Ethiopia, 2003<br>(LMIC)	Cross-sectional<br>305 (253 analyzed; n=92 stunted; 161 nonstunted)	1 collection time between 5-11 mo	1 sampling time between 5-11 mo	Concentration in HM<br>2.2 ± 0.1 (SEM) µmol/L (unstunted)	stunting<br>(NCHS)	(No) Associations among milk copper and stunting	Infant age
**Chromium**							
Motoyama et al.<br>Japan, 2021<br>(HIC)	Cohort<br><br>129 enroleed (79 analysed)	1 mo and 3 mo	1 mo and 3 mo	Concentration in HM	weight, height, HC<br>(no standards)	(No) Association between Cr and infant length, weight or HC	None
**Iodine**							
Bilston-John et al,<br>Australia, 2021<br>(HIC)	Cohort<br>83	1, 2, 3, 4, 5, 6, 9, and 12 mo postpartum	3 and 6 mo postpartum	Concentration and estimated intake	weight, length, HC, LFAZ, HCFAZ, WFAZ<br>(WHO Standards)	(No) Association between any micro or macro minterals and infants anthropometrics	None
Ellsworth et al.<br>USA, 2020<br>(HIC)	Longitudinal<br>57 (35 included in analyses)	2 wks	2 wks, 2 mo, 6 mo, and 1 year.	Concentration in HM<br>Data not extractable	WFAZ, LFAZ, WFLZ	(+) Associations between iodine and WFA, WFLZ<br>(No) Association between iodine and LFAZ	**(Maternal):** maternal BMI (assumed to be pre-pregnancy), interaction of maternal BMI and time;<br>**(Infant)** birth anthropometric Z-score, gender
Nazeri et al.<br>Iran, 2020<br>(LMIC)	Longitudinal<br>94	3-5 days postpartum	6 mo	Concentration in HM<br>232.5 (157.5, 296.0) µg/L	WAZ, LAZ, WFLZ, HCAZ	(+) Associations between iodine and WFLZ<br>(No) Associations between iodine and WAZ, LAZ, HCAZ	Maternal age, educational level, pre-pregnancy maternal BMI, parity, type of feeding at 6 mo and birth anthropometric measurements; Also reported IGF-1, adiponectin, leptin
**Selenium (Se)**							
Bilston-John et al,<br>Australia, 2021<br>(HIC)	Cohort<br>83	1, 2, 3, 4, 5, 6, 9, and 12 mo postpartum	3 and 6 mo postpartum	Concentration and estimated intake	weight, length, HC, LFAZ, HCFAZ, WFAZ<br><br>(WHO Standards)	(No) Association between any micro or macro minterals and infants anthropometrics	None
Han et al.<br>China, 2021<br>(LMIC)	Cross-sectional<br>305<br>253 analysed (92 in stunted group; 161 non-stunted group_	42 days postpartum	42 days postpartum	Concentration in HM	Stunting<br>(WHO standard)<br>LAZ <-2 (stunding)<br>WAZ <-2 (underweight)<br>WLZ = -2 (wasting)<br>WLZ = 2 (overweight)<br>WAZ >2 (overweight)<br>WLZ >3 (obesity)	(-) Assocation between HM Se and infant WLZ<br>(no) Association between HM Se and infant LAZ or infant WAZ	Daily dietary intake of total energy and three macronutrients and household income, age, parity, Z-scores at birth
Li et al.<br>Guatemala, 2019<br>(LMIC)	Longitudinal<br>114;<br>80 Mothers without subclinical mastitis (non-SCM) during early lactation (2-46 days)<br>34 with SCM in early lactation (2-46 days)<br>103 non-SCM in established lactation (4-6 mo)<br>11 with SCM in established lactation (4-6 mo)	Early (2-46 days) and established lactation (4-6 mo)	Early (2-46 days) and established lactation (4-6 mo)	Concentration in HM<br>0.18 ± 0.03 µmol/L (early, non-SCM)<br>0.22 + 0.08 (early, SCM)<br>0.14 ± 0.03 (established, non-SCM)<br>0.18 ± 0.06 (established, SCM)	WAZ, LAZ, WLZ, HCAZ,at early lactation and with growth velocity (change in infant growth/d) for weight, length, and HC between early and established lactation	(No) Assumed associations between Se and WAZ, LAZ, WLZ, HCAZ, or growth velocity	**Early lactation models:** somatic cell count, milk cytokine concentrations, estimated milk intake<br><br>**Growth trajectory models:** variables from early lactation models, plus infant sex, early length or HC, maternal weight, height, and mixed feeding (yes/no)
Motoyama et al.<br>Japan, 2021<br>(HIC)	Cohort<br><br>129 enroleed (79 analysed)	1 mo and 3 mo	1 mo and 3 mo	Concentration in HM	weight, height, HC<br>(no standards)	(No) Association between Se and infant length, weight or HC	None
Nikniaz et al.<br>Iran, 2019<br>(LMIC)	Cross-sectional<br>57;<br>30 in synbiotic group<br>27 in placebo group	Unclear; Before intervention	Unclear; Before intervention	Concentration in HM<br>48.5 ± 12.1 µg/L (baseline, placebo)<br>46.5 ± 13.6 µg/L (30 d later, placebo)	WAZ, HAZ	(+) Associations between high breast milk Se and WAZ, HAZ	None
**Manganese (Mn)**							
Bilston-John et al,<br>Australia, 2021<br>(HIC)	Cohort<br>83	1, 2, 3, 4, 5, 6, 9, and 12 mo postpartum	3 and 6 mo postpartum	Concentration and estimated intake	weight, length, HC, LFAZ, HCFAZ, WFAZ<br><br>(WHO Standards)	(No) Association between any micro or macro minterals and infants anthropometrics	None

Table S3. Characteristics and results of included studies reporting on human milk micronutrients and infant anthropometrics - organized by nutrient.

Authors, country, publication year (income setting)	Design and participants	Timing of milk sampling	Timing of infant anthropometrics	Estimated intake or HM concentration*	Anthropometric outcome measures and standards	Associations**	Major confounders considered
Li et al.<br>Guatemala, 2019<br>(LMIC)	Longitudinal<br>114;<br>80 Mothers without subclinical mastitis (non-SCM) during early lactation (2-46 days)<br>34 with SCM in early lactation (2-46 days)<br>103 non-SCM in established lactation (4-6 mo)<br>11 with SCM in established lactation (4-6 mo)	Early (2-46 days) and established lactation (4-6 mo)	Early (2-46 days) and established lactation (4-6 mo)	Concentration in HM<br>0.17 ± 0.12 µmol/L (early, non-SCM)<br>0.20 ± 0.30 (early, SCM)<br>0.13 ± 0.10 (established, non-SCM)<br>0.16 ± 0.14 (established, SCM)	WAZ, LAZ, WLZ, HCAZ,at early lactation and with growth velocity (change in infant growth/d) for weight, length, and HC between early and established lactation	(+) Associations between Mn and WAZ<br>(No) Assumed associations between Mn and LAZ, WLZ, HCAZ, or growth velocity	**Early lactation models:** somatic cell count, milk cytokine concentrations, estimated milk intake<br><br>**Growth trajectory models:** variables from early lactation models, plus infant sex, early length or HC, maternal weight, height, and mixed feeding (yes/no)
Motoyama et al.<br>Japan, 2021<br>(HIC)	Cohort<br>129 enroleed (79 analysed)	1 mo and 3 mo	1 mo and 3 mo	Concentration in HM	weight, height, HC<br>(no standards)	(No) Association between Mn and infant length, weight or HC	None
**Multiple HM analytes**							
Li et al.<br>Guatemala, 2016<br>(LMIC)	234;<br>56 transitional milk samples (5-17 d);<br>75 early milk samples (18-46 d);<br>103 established milk samples (4-6 mo)	Cross-sectional, 3 groups:<br>Early (5-17 d)<br>Transitional (28-46 d)<br>Established (4-6 mo)	Same as milk sampling, cross-sectional.	Multivariable analyses of: Ca, Cu, Fe, Mg, Mn, K, Rb, Se, Na, Sr, Zn	WAZ, LAZ, HCAZ	(+) Associations between multiple micronutrients and WAZ, LAZ, and HCAZ during early and established lactation, but not transitional lactation	Infant sex, maternal height, and shipment

*Values reported as mean ± SD or median (IQR). **"No (assumed) associations = unreported associations assumed to be no association.

Abbreviations: HIC, high income countries; mo, months; HM, human milk; LMIC, low and middle income countries; NCHS, National Center for Health Statistics; SCM, subclinical mastitis; WHO, World Health Organization; wks, weeks

Anthropometrics: BMI, body mass indez; HAZ, height for age z-score; HC, head circumference; HCAZ, head circumference z-score; LAZ, length for age Z-score; WAZ, weight for age z-score; WLZ, weight-for-length z-score

Elements: Ca, calcium; Cu, copper; Fe, iron; K, potassium; P, phosphorus; Mg, magnesium; Mn, manganese; Na, sodium; Rb, rubidium; Se, selenium; Sr, strontium; Zn, zinc
